# Targeted degradation of a constitutively active GPCR oncoprotein

**DOI:** 10.1016/j.isci.2026.118116

**Published:** 2026-11-05

**Authors:** Victoria R. Saca, Yuanhuang Chen, Colin Burdette, Thomas Huber, Thomas P. Sakmar

**Affiliations:** 1 Laboratory of Chemical Biology and Signal Transduction, The Rockefeller University, New York, NY 10065, USA; 2 Tri-Institutional PhD Program in Chemical Biology, New York, NY 10065, USA

**Keywords:** G protein-coupled receptor, GPCR, proteolysis-targeting chimeras, PROTAC, targeted protein degradation, uveal melanoma, CysLTR2

## Abstract

To assess the feasibility of using proteolysis-targeting chimeras (PROTACs) to target G protein-coupled receptors (GPCRs), we constructed a degron-tagged GPCR library in which a HaloTag7 protein fusion tag was engineered into the cytoplasmic carboxyl terminal tail. We used luciferase complementation and immunoblot assays to quantify cellular receptor protein levels after treatment with a von Hippel-Lindau (VHL) E3 ligase-recruiting bifunctional molecule, HaloPROTAC3. HaloPROTAC3 treatment resulted in recruitment of the VHL E3 ligase, yielding a time- and dose-dependent decrease in receptor expression and decreased agonist-dependent receptor signaling. We then focused on human cysteinyl leukotriene receptor 2 (CysLTR2). The CysLTR2 mutant L129Q constitutively signals through heterotrimeric G proteins and drives tumorigenesis in uveal melanoma (UM). We observed a HaloPROTAC3 dose-dependent decrease in receptor levels in primary human melanocytes expressing the engineered CysLTR2-L129Q mutant. Our results support the potential utility of using PROTACs as GPCR degraders.

## Highlights

• A modular degron library enables targeted destruction of cell surface receptors
• Chimeric degraders selectively deplete active oncogenic mutant receptors
• Receptors bearing an activating tumor mutation undergo proteasomal breakdown
• Oncoprotein levels are successfully lowered in primary human skin melanocytes

## Graphical abstract

**Figure:**
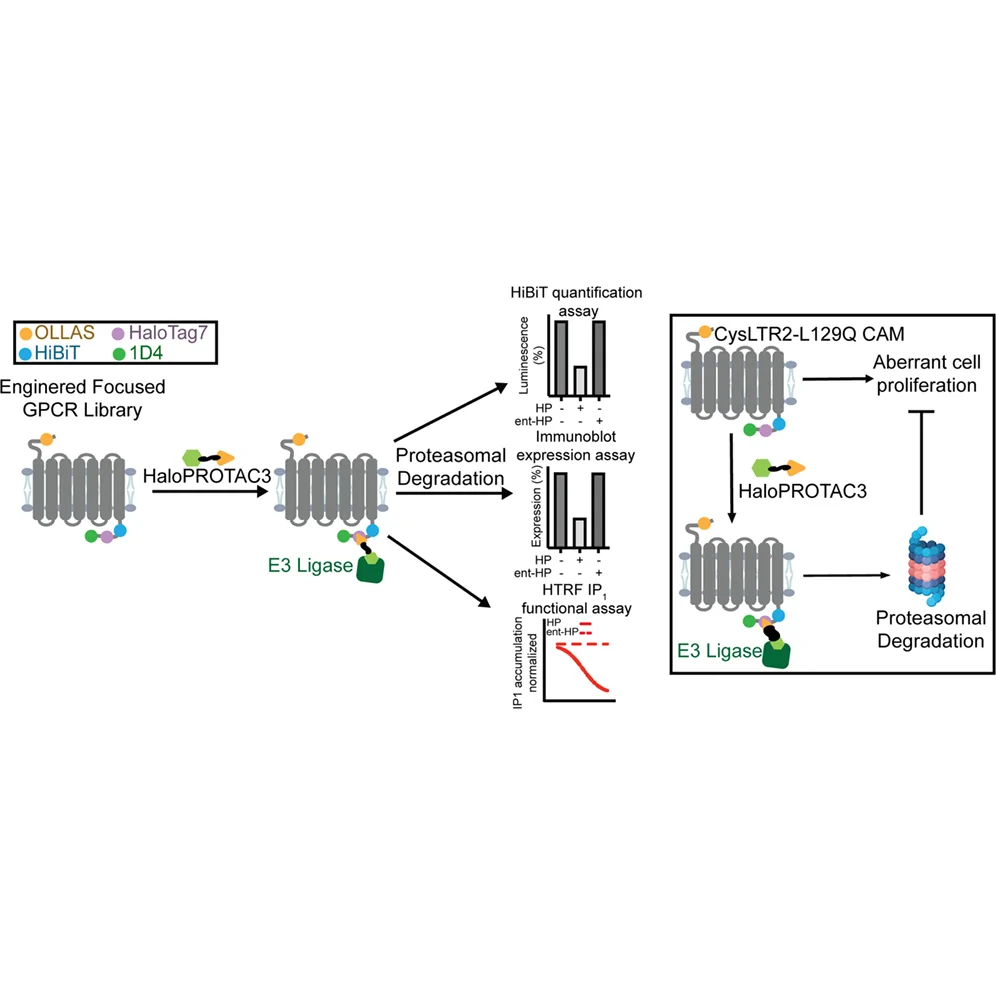

## Teaser

Pharmacology; Molecular biology; Cancer

## Introduction

G protein-coupled receptors (GPCRs) comprise a family of transmembrane signaling proteins involved in various cellular processes, including homeostasis, cell growth, and proliferation. The binding of a specific agonist ligand induces a conformational change in the receptor that alters its association with a heterotrimeric guanine nucleotide-binding regulatory protein (G protein), which in turn activates downstream cellular signaling cascades. Dysregulation of GPCRs or G protein signaling, including constitutive signaling, has been associated with the development and progression of cancer.^1^^,^^2^^,^^3^ Specifically, aberrant GPCR and G protein signaling due to overexpression or constitutive activity has been associated with breast cancer,^4^^,^^5^^,^^6^^,^^7^ prostate cancer,^8^^,^^9^ and basal cell carcinoma.^10^^,^^11^^,^^12^ Uveal melanoma (UM) is the most common primary malignant tumor of the eye. Most cases of UM are caused by activating mutations in the genes *GNAQ* or *GNA11*, which encode the α subunits of Gα_q_ and Gα_11_, respectively.^13^^,^^14^ Notably, about 3% of UM cases are caused by an activating mutation in the gene *CYSLTR2*, which encodes the upstream GPCR cysteinyl leukotriene receptor 2 (CysLTR2).^15^ The *CYSLTR2* oncogene harbors a missense point mutation causing the substitution of Leu with Gln at position 129, resulting in receptor activation even in the absence of an agonist ligand.^16^^,^^17^ CysLTR2-L129Q displays a gain-of-function (GoF) constitutively active mutant (CAM) phenotype with respect to G protein signaling. However, the CysLTR2 CAM recruits β-arrestin very poorly and escapes cellular downregulation mechanisms.^16^

GPCRs are considered desirable drug targets because of their location in the plasma membrane, providing extracellular access for drugs that can modulate diverse downstream signaling cascades. About 30% of all FDA-approved drugs target GPCRs, however, only about 150 unique GPCRs out of a family of ∼400 non-olfactory members have been targeted to date.^18^^,^^19^ Only a few of these drugs target GPCRs as cancer therapeutics, creating a gap in therapeutic opportunities.^2^^,^^20^ An ideal drug candidate for the CysLTR2 CAM oncoreceptor might be an inverse agonist small molecule that could turn off its constitutive signaling. However, despite significant screening efforts, no such lead compound has been reported, and the known CysLTR2 antagonists BayCysLT2 and HAMI3379 show no inverse agonist activity at CysLTR2-L129Q.^21^

Targeted protein degradation (TPD) provides opportunities for cancer therapeutics when GPCR overexpression or a GPCR CAM drives cancer progression. For example, proteolysis-targeting chimeras (PROTACs) have been reported to decrease the abundance of aberrant proteins.^22^^,^^23^ PROTACs are heterobifunctional molecules where one functional moiety targets the protein of interest, and the other functional moiety recruits an E3 ligase.^24^^,^^25^ PROTACs ideally facilitate ubiquitin transfer to the target protein, which tags it for proteasomal degradation.^25^ The PROTAC strategy has been applied to multiple soluble proteins; however, only recently has this TPD drug discovery platform been applied to membrane proteins such as GPCRs. Ubiquitination of GPCRs has been associated with various outcomes, including internalization, trafficking, and both lysosomal and proteasomal degradation.^26^^,^^27^^,^^28^ A few receptors associated with proteasomal degradation include the dopamine 4 receptor (D4R),^29^ metabotropic glutamate receptor 1 and 5 (mGluR1a and mGluR5),^30^ the μ and δ opioid receptors,^31^ and the thyrotropin-releasing hormone receptor.^32^ Three PROTACs have been reported to target GPCRs for proteasomal degradation; however, the degradation mechanism remains unclear for two GPCR-targeting PROTACs due to the extracellular orientation of the E3 ligase-recruiting ligand.^33^^,^^34^^,^^35^^,^^36^^,^^37^ These studies using so-called conditional degron tags (CDTs) and specific PROTACs provide a starting point demonstrating the feasibility of PROTACs as a possible drug discovery platform for controlled proteasomal degradation of GPCRs.^38^^,^^39^ Alternative TPD methods, such as LYTACs, which consist of an antibody to the POI and a lysosome-targeting moiety to shuttle proteins to the lysosomal degradation pathway, could be used to degrade GPCRs. However, there is no equivalent CDT technology available for LYTACs to facilitate multi-receptor screening in a proof-of-concept study. Additionally, recently a PROTAC-like heterobifunctional molecular degrader was created for the CC chemokine receptor 2 (CCR2). This degrader binds to at an intracellular allosteric site and promotes the lysosomal degradation of the receptor, indicating that PROTAC technology and molecular design could be applied for both proteasomal and lysosomal degradation.^40^

Here, we further validate the use of PROTACs using a focused GPCR library of Gα_q_-coupled receptors through protein levels and downstream signaling as a proof-of-concept. We created a focused GPCR library with a C-terminal CDT to promote the intracellular orientation of a PROTAC, thereby promoting E3 ligase recruitment, ubiquitination, and degradation. We show that these GPCRs are possible targets for PROTACs and provide evidence for using an initial CDT screen to determine feasible GPCR targets for PROTAC development. Additionally, we expanded this methodology for a known oncogenic GPCR, CysLTR2-L129Q. HaloPROTAC3 treatment reduced protein abundance and signaling of CysLTR2-L129Q, showing that PROTACs can inhibit constitutive activity. Therefore, this work explores the possibility of using the PROTAC drug discovery platform for GPCR and G protein CAMs as therapeutic options.

## Results

We first optimized a low-efficiency transient expression system for expression of engineered GPCRs tagged with conditional degrons. We designed CysLTR2 constructs with a HiBiT tag and a HaloTag7 on the cytoplasmic C-terminal tail of the receptor. The HaloTag7 serves as a binding site for the HaloPROTAC3 ligand (Figure S1). The HiBiT tag enables the quantification of receptor expression via a luminescence-based nanoluciferase protein complementation assay, referred to as the HiBiT quantification assay.^41^^,^^42^ We then compared receptor expression under a weak HSV-TK promoter versus a strong CMV promoter (Figures 1A and S2).^43^ Additionally, we used a secondary messenger d-myo-inositol-1-phosphate (IP_1_) accumulation assay to measure the changes in activation of the Gα_q_ signaling pathway. This assay is a competitive homogeneous time-resolved fluorescence (HTRF) assay where a d2-labeled IP_1_ acts as the fluorescence acceptor and a Terbium cryptate-labeled monoclonal antibody acts as the fluorescence donor. Lithium chloride (LiCl) is used to inhibit the degradation of IP_1_ by inositol monophosphates (IMPase). The energy transfer measured in the assay at discrete time points is thus related to the concentration of IP_1_ and provides information about the activity of the Gα_q_ signaling pathway. Notably, this assay can detect constitutive activity in GPRCs due to the accumulation of IP1 providing a sustained signal. For the CysLTR2 low- and high-expressing constructs we saw a ∼70% decrease in overall signaling amplitude for the HSV-TK weak promoter system and a right shift in the potency of the agonist ligand as compared with a CMV promoter reference construct (Figures 1B and 1C).^16^ We quantified the overall protein levels using the HiBiT quantification assay and saw ∼7,000 counts in luminescence for the HSV-TK weak promoter construct, while the CMV strong promoter construct had a ∼250,000 counts in luminescence (Figures 1D and S2). Additionally, we confirmed the specific binding of the HaloPROTAC3 to the HaloTag7, as a reference construct without the C-terminal HaloTag7 showed no decrease in signaling with increasing concentrations of HaloPROTAC3 (Figure S2).

**Figure 1:**
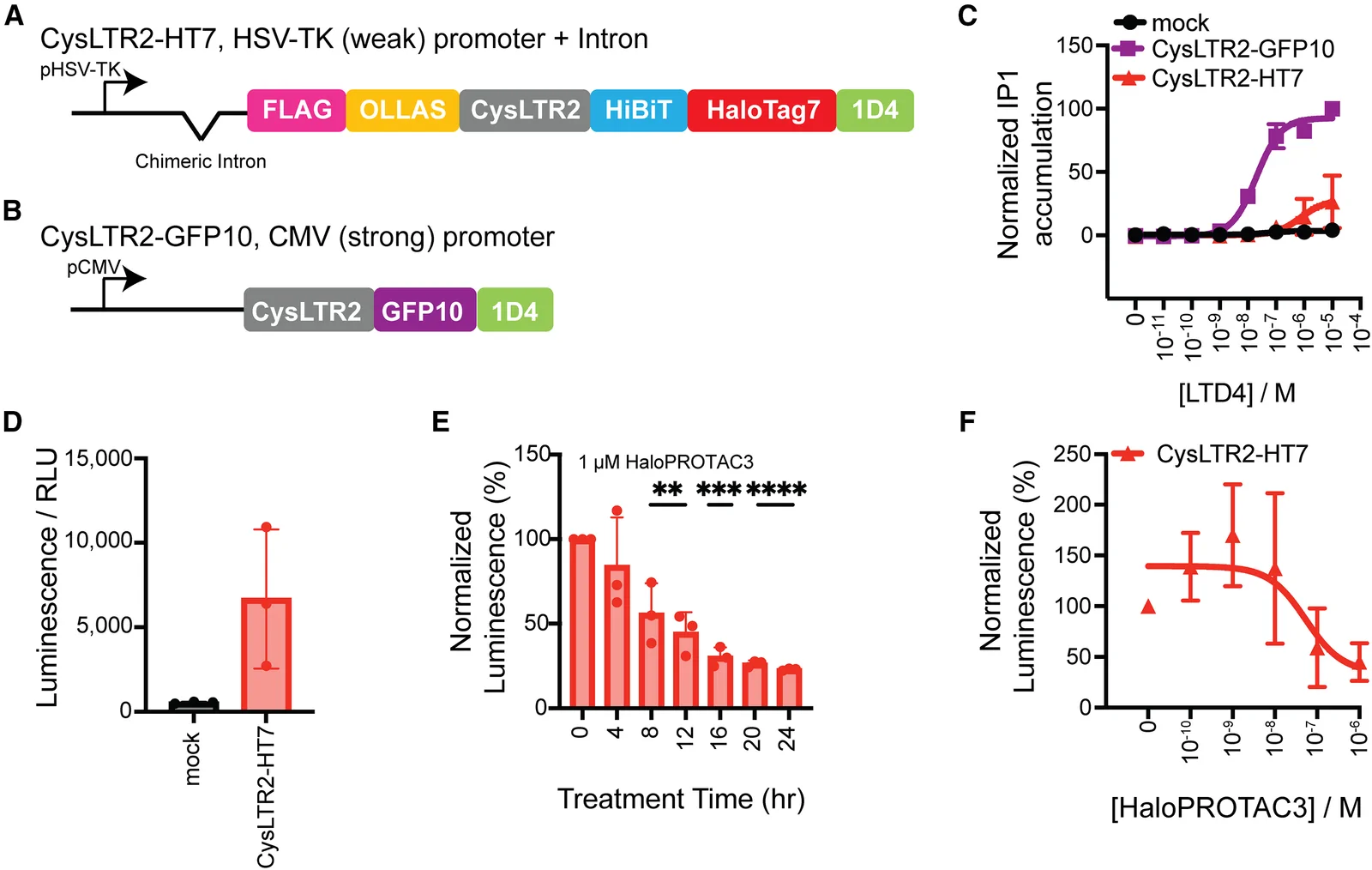
Characterization of engineered GPCR expression constructs (A) GPCR expression constructs with two N-terminal epitope tags (FLAG and OLLAS), C-terminal bioluminescent nanoluciferase protein complementation tag (HiBiT), an engineered HaloTag7, and an epitope tag (1D4) under a weak promoter (HSV-TK) were prepared for ten GPCRs. The CysLTR2 construct is used here as an example. (B) CysLTR2 with a C-terminal green fluorescent protein 10 (GFP10) and epitope tag (1D4) was used as a reference construct under the CMV promoter. (C) A second messenger inositol monophosphate (IP_1_) accumulation assay showed agonist dose-dependent IP_1_ accumulation. Data were normalized to the highest concentration of agonist of the reference construct (CysLTR2-GFP10). Data were then plotted and fit to a sigmoidal or horizontal line model and compared using the Akaike Information Criterion. Each data point represents the mean signal ±SD of three individual experiments. (D) HiBiT complementation luminescence assay was carried out using the weak promoter expression construct CysLTR2-HT7. Each data point represents the mean ± SD signal from three technical replicates. Normalized data were compared to the condition without treatment using a one-way ANOVA with multiple comparisons. (E) Addition of HaloPROTAC3 to cells expressing CysLTR2-HT7 caused a time-dependent decrease in HiBiT complementation luminescence. Data were normalized to the condition without HaloPROTAC3. Significance bars indicate conditions with the same significance as compared to the condition without HaloPROTAC3. Each point represents the mean ± SD of normalized data from three technical replicates. Statistical significance is indicated as follows: ns, *p* > 0.05; ∗, *p* ≤ 0.05; ∗∗, *p* ≤ 0.01; ∗∗∗, *p* ≤ 0.001; and ∗∗∗∗, *p* ≤ 0.0001. (F) Addition of HaloPROTAC3 to cells expressing CysLTR2-HT7 caused a dose-dependent decrease in HiBiT complementation luminescence. Data were normalized to the receptor condition without treatment and plotted and fit to a three-parameter inhibitor response or horizontal line model and compared using Akaike’s Information Criterion. Each point represents the mean ± SD of normalized data from three individual experiments.

We then tested the effects of HaloPROTAC3 on the HSV-TK weak promoter construct. We transfected HEK293T cells with the HSV-TK weak promoter construct and allowed for 24 h of protein expression. We observed a time-dependent decrease in the overall protein levels when treating cells transfected with the HSV-TK weak promoter construct with 1 μM HaloPROTAC3. We saw ∼50% decrease in luminescence at around 8 h of HaloPROTAC3 treatment. At 16 hours of treatment, the decrease in protein level starts to plateau at ∼70% decrease (Figure 1E). We next tested various concentrations of HaloPROTAC3 for 16 h. We saw a ∼50% decrease in total protein levels at a concentration of 1 μM HaloPROTAC3 (Figure 1F). The CMV promoter strong construct, had no decrease in overall protein levels with various concentrations (Figure S2).

### Focused GPCR library is degraded by HaloPROTAC3

Using a pool of 210 expression-validated GPCR constructs from the DuET library,^44^ we designed and created a focused library of Gα_q_-coupled GPCR expression constructs using a weak HSV-TK promoter system, including the HiBiT tag and a HaloTag7 on the cytoplasmic C-terminal tail of the receptor. Initially, we determined all Gα_q_ -coupled receptors with commercially available agonist ligands within the DuET library. We then performed an initial IP1 accumulation assay to assess the activation of the DuET library construct with commercially available agonist ligands. The receptors that showed dose-dependent activation were then cloned in the HSV-TK weak promoter expression construct backbone to create the final GPCR expression library construct. Each receptor in the library shows activation of the Gα_q_ signaling pathway upon addition of the specific agonist ligand. (Figure S3). We then evaluated the effects of HaloPROTAC3 and an inactive enantiomer, ent-HaloPROTAC3, on the protein levels and signaling of each receptor. HEK293T cells were transiently transfected with the engineered constructs, enabling protein expression for 24 h before adding HaloPROTAC3 or ent-HaloPROTAC3. Following treatment, we performed the HiBiT luciferase complementation assay (Figure S4) and immunoblot analysis, where we probed the C-terminal HiBiT tag or the N-terminus of the receptor, respectively, to show protein abundance (Figures 2A and S4). The HiBiT quantification assay showed decrease in receptor luminescence for many receptors. Specifically, two receptors (CysLTR1, MRGPRX1) showed a ∼70–80% reduction and seven receptors showing a ∼50–60% reduction (CysLTR2, HRH1, MRGPRX4, ADRA1A, LPAR5, P2RY4, CHRM1). The P2RY2 receptor showed no significant reduction in luminescence when incubated with the HaloPROTAC3 (Figure S4). The addition of ent-HaloPROTAC3 resulted in a restoration of luminescence for several receptors, including two receptors with a ∼30–40% rescue of luminescence (MRGPRX1, MRGPRX4) and four receptors with a ∼20% rescue of luminescence (CysLTR1, CysLTR2, HRH1, CHRM1) (Figure S4). On the other hand, the immunoblot shows a ∼50–60% reduction with the addition of the HaloPROTAC3 for CysLTR2 and a ∼50–60% decrease in expression for CysLTR1 (Figure 2A). Additionally, we saw a ∼70–80% reduction in five receptors (HRH1, MRGPRX1, MRGPRX4, P2RY2, CHRM1) and three receptors show a ∼90% reduction (LPAR5, ADRA1A, P2RY4) (Figure 2A). All receptors show full rescue or higher expression levels with the addition of the enantiomer HaloPROTAC3 in the immunoblot.

**Figure 2:**
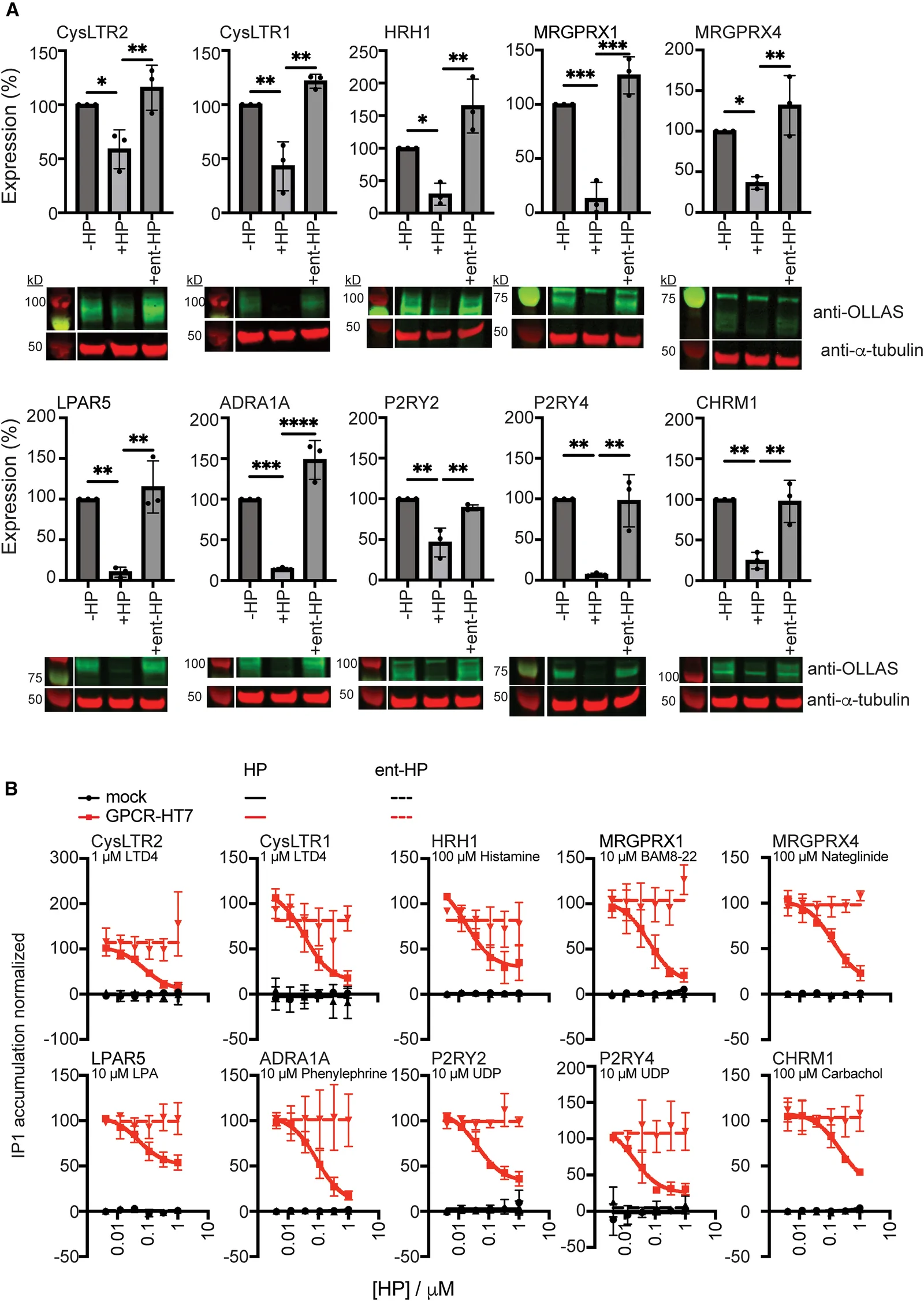
HaloPROTAC3 degrades ten different GPCR degron constructs (A) Immunoblot analysis after treatment with HaloPROTAC3 (HP) or enantiomer (ent-HP). The immunoblots shown for each GPCR are representative images from three independent experiments. Data were normalized and compared to the condition without treatment using a one-way ANOVA with multiple comparisons. Each point represents data from one individual experiment. Data were compared to the condition without HaloPROTAC3 using a one-way ANOVA with multiple comparisons. Statistical significance is indicated as follows for all graphs: ns, *p* > 0.05; ∗, *p* ≤ 0.05; ∗∗, *p* ≤ 0.01; ∗∗∗, *p* ≤ 0.001; and ∗∗∗∗, *p* ≤ 0.0001. (B) IP_1_ accumulation assay after treatment with HP or ent-HP and an agonist specific for each GPCR. Data were plotted and fit to a three-parameter response or horizontal line model and compared using Akaike’s Information Criterion. Each data point represents the mean signal ±SD from three independent experiments.

The immunoblot results indicated that HaloPROTAC3 reduces the protein levels of several GPCRs. To validate these results, we tested the effects of the HaloPROTAC3 on receptor signaling of the receptors using a functional assay, the HTRF IP_1_ immunoassay. The HTRF IP_1_ immunoassay measures the accumulation of inositol monophosphate (IP_1_), a secondary messenger in the Gα_q_ signaling pathway. Similarly, we transiently transfected HEK293T cells with individual receptor constructs, and after 24 h of protein expression, we incubated them with varying concentrations of HaloPROTAC3 and ent-HaloPROTAC3. The assay was then conducted after a 2-h incubation with the agonist-ligand. Increasing concentrations of the HaloPROTAC3 dose-dependently decreased the IP_1_ accumulation for each receptor (Figure 2B). Overall, we observed at least a ∼50% decrease in IP_1_ accumulation for each receptor (Figure 2B). Although there was efficacy across all receptors, the efficacies varied, with IC50 values ranging from 0.0179 μM (HRH1) to 0.2268 μM (CHRM1) (Table S5). On the other hand, increasing concentrations of the ent-HaloPROTAC3 did not decrease the IP_1_ accumulation in any of the receptors (Figure 2B).

### CysLTR2-L129Q undergoes proteasomal degradation in response to HaloPROTAC3

The results from studies of the focused GPCR library suggested that HaloPROTAC3 could decrease the receptor protein levels as well as the agonist-dependent IP_1_ accumulation of the GPCRs tested. Therefore, we tested whether the HaloPROTAC3 strategy could affect the protein level and signaling of the oncogenic mutant CysLTR2-L129Q. HEK293T cells were transfected with either CysLTR2-WT or CysLTR2-L129Q and then treated with various concentrations of HaloPROTAC3 and a concentration of ent-HaloPROTAC3. The protein expression was then visualized through immunoblotting. Through immunoblot analysis, we saw a ∼60% degradation of CysLTR2-WT at high concentrations of HaloPROTAC3, which is consistent with previous results from the focused library (Figure 3A). Additionally, treatment of the ent-HaloPROTAC3 did not affect the protein abundance signal of CysLTR2-WT (Figure 3A). For CysLTR2-L129Q, we saw a ∼50% decrease in signal with 1 μM of HaloPROTAC3 (Figure 3A). Treatment with ent-HaloPROTAC3 also showed full rescue in protein abundance (Figure 3A). We next performed the IP_1_ accumulation assay to determine the effects of the HaloPROTAC3 and ent-HaloPROTAC3 on agonist-dependent and agonist-independent signaling. Cells transfected with CysLTR2-WT or the constitutively active CysLTR2-L129Q mutant were tested both with and without the agonist ligand, LTD4, in addition to HaloPROTAC3 or ent-HaloPROTAC3. CysLTR2-WT showed a decrease in IP_1_ accumulation of ∼50% in the presence of high concentrations of HaloPROTAC3 with LTD4. Full signaling was observed after treatment with ent-HaloPROTAC3, as previously seen in experiments with the focused receptor library (Figure 3B). CysLTR2-WT without LTD4 did not show signaling; therefore, there was no effect of HaloPROTAC3 or ent-HaloPROTAC3 (Figure 3B). For CysLTR2-L129Q cells treated with LTD4, there was a ∼40% decrease in IP1 accumulation at high concentrations, and for CysLTR2-L129Q cells without LTD4, there was a ∼50% decrease in IP1 accumulation compared with the untreated signal (Figure 3B). Additionally, there was a rescue of the constitutive IP_1_ accumulation when both conditions of CysLTR2-L129Q were treated with ent-HaloPROTAC3. (Figure 3B).

**Figure 3:**
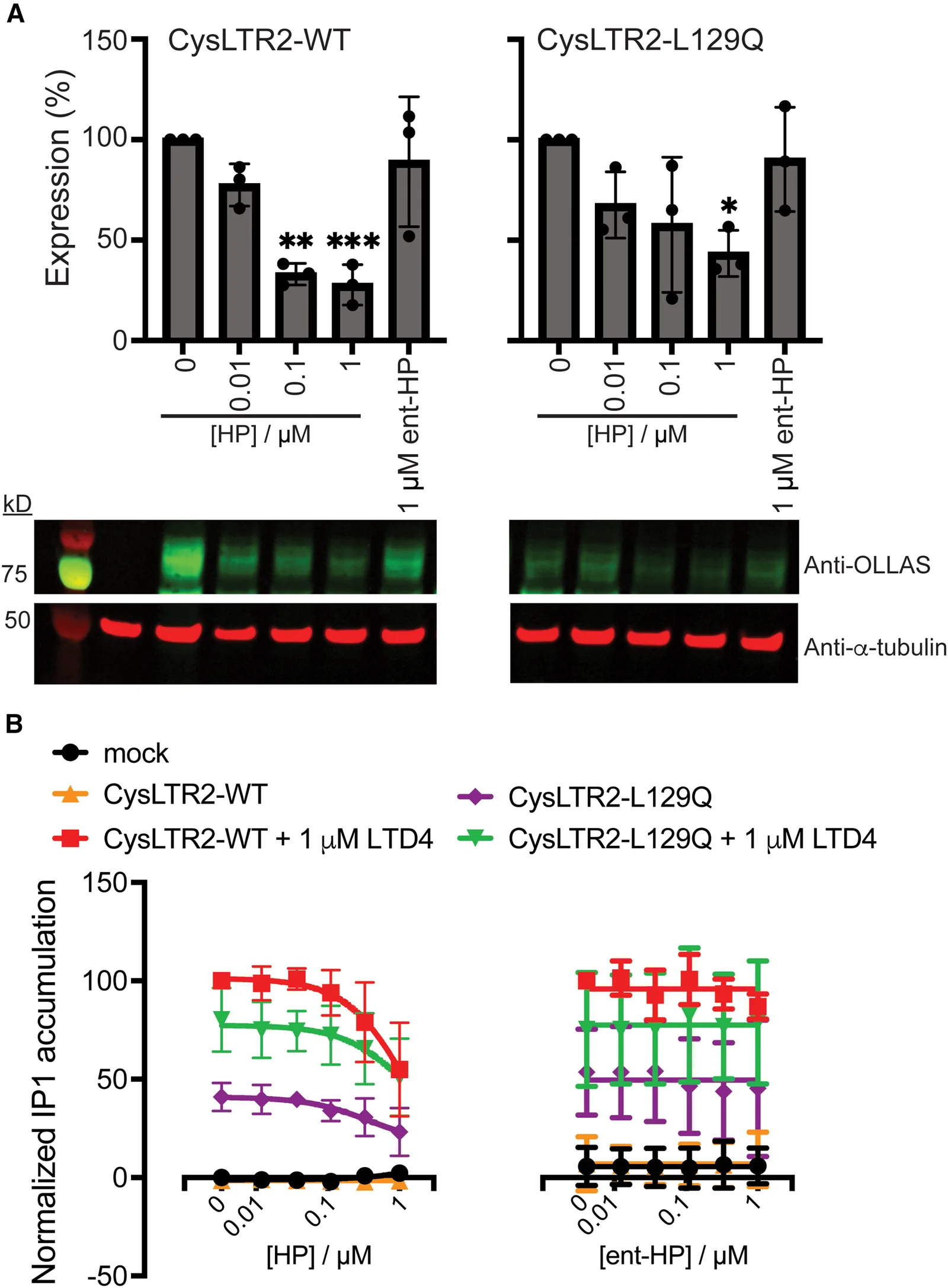
HaloPROTAC3 decreases the expression and signaling of CysLTR2-WT and CysLTR2-L129Q in a dose-dependent manner (A) Quantification of immunoblot signal of CysLTR2-WT and CysLTR2-L129Q after treatment with HaloPROTAC3 (HP) or the inactive enantiomer, ent-HaloPROTAC3 (ent-HP). The immunoblots are representative images from three independent experiments. Data were normalized and compared to the condition without HP using multiple comparisons and one-way ANOVA. Each data point represents the signal from one individual experiment. Statistical significance is indicated as follows for all graphs: ns, *p* > 0.05; ∗, *p* ≤ 0.05; ∗∗, *p* ≤ 0.01; ∗∗∗, *p* ≤ 0.001; and ∗∗∗∗, *p* ≤ 0.0001. (B) IP_1_ accumulation after treatment with various concentrations of HP or ent-HP, and with or without agonist. Data were plotted and fit to a three-parameter response or horizontal line model and compared using Akaike’s Information Criterion. Each data point represents the mean signal ±SD from three independent experiments.

We next performed competition assays to confirm formation of a ternary complex between the HaloPROTAC3, CysLTR2-L129Q, and the E3 ligase. We used the IP_1_ accumulation assays as a readout of signal, since the therapeutic effect of a potential PROTAC for CysLTR2-L129Q would be to decrease constitutive signaling. We used a VHL ligand, VL285, to compete with the HaloPROTAC3. For CysLTR2-WT and CysLTR2-L129Q with and without agonist, we saw a ∼50–60% decrease in IP_1_ accumulation with the addition of 1 μM HaloPROTAC3 alone, confirming previous results. Additionally, when only adding VL285, there was no significant difference in IP_1_ accumulation as compared to the vehicle condition in CysLTR2-WT and CysLTR2-L129Q in the presence and absence of LTD4. In conditions with CysLTR2-WT, when VL285 was added as a competitor of HaloPROTAC3, there was a rescue of IP_1_ accumulation. Additionally, we saw a similar rescue for CysLTR2-L129Q-mediated IP_1_ accumulation both in the presence and absence of LTD4 (Figure 4A). To test for engagement of the other end of the HaloPROTAC3 molecule, which binds to the HaloTag7 on the receptor, we treated with the substrate competitor 7-bromo-1-heptanol.^45^ Again, we first saw a significant decrease (∼40–50%) in IP_1_ accumulation with the addition of the HaloPROTAC3 alone. The addition of 7-bromo-1-heptanol alone did not significantly affect the IP_1_ accumulation in any condition. The addition of 7-bromo-1-heptanol as a competitor for covalent binding of the HaloPROTAC3 molecule to the HaloTag7 on the engineered receptors showed rescue in IP_1_ accumulation for CysLTR2-WT and CysLTR2-L129Q in both the presence and absence of LTD4 (Figure 4B).

**Figure 4:**
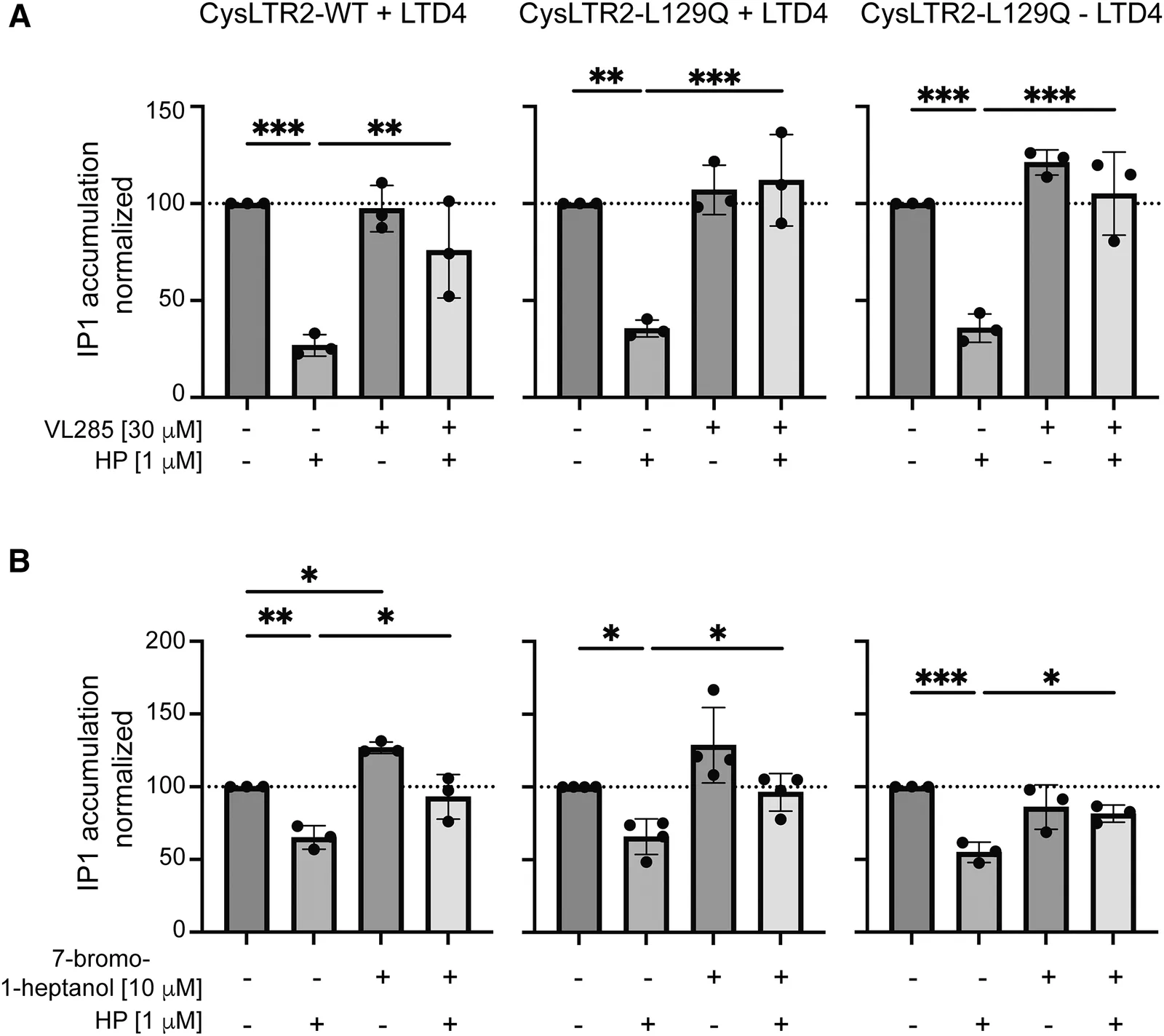
Evidence for the formation of a ternary complex between CysLTR2-WT/CysLTR2-L129Q, HaloPROTAC3, and E3 ligase (A) IP_1_ accumulation after pretreatment with VL285 and treatment with HaloPROTAC3 (HP). (B) IP_1_ accumulation after pretreatment with 7-bromo-1-heptanol and treatment with HP. Each data point represents the mean signal ±SD from three technical replicates. Conditions were compared using a one-way ANOVA with multiple comparisons. Statistical significance is indicated as follows for all graphs: ns, p > 0.05; ∗, p ≤ 0.05; ∗∗, p ≤ 0.01; ∗∗∗, p ≤ 0.001; and ∗∗∗∗, p ≤ 0.0001.

We then aimed to understand whether the mechanism of the decrease in expression and signaling of CysLTR2-WT and CysLTR2-L129Q was due to activation of the ubiquitin-proteasome degradation pathway. We used MLN4924, a NEDD8-activating enzyme (NAE) inhibitor, which inhibits neddylation and activation of Cullin-RING ligases, such as the VHL E3 ligase. Treatment with MLN4924 alone resulted in significantly higher IP_1_ accumulation for CysLTR2-WT and CysLTR2-L129Q in the absence of ligand (Figure 5A). As previously shown, HaloPROTAC3 treatment caused a consistent ∼50–60% decrease in IP_1_ accumulation for all receptor conditions. When HaloPROTAC3 was added in the presence of MLN4924, there was a full restoration of IP_1_ accumulation for CysLTR2-WT and CysLTR2-L129Q in the presence and absence of LTD4.

**Figure 5:**
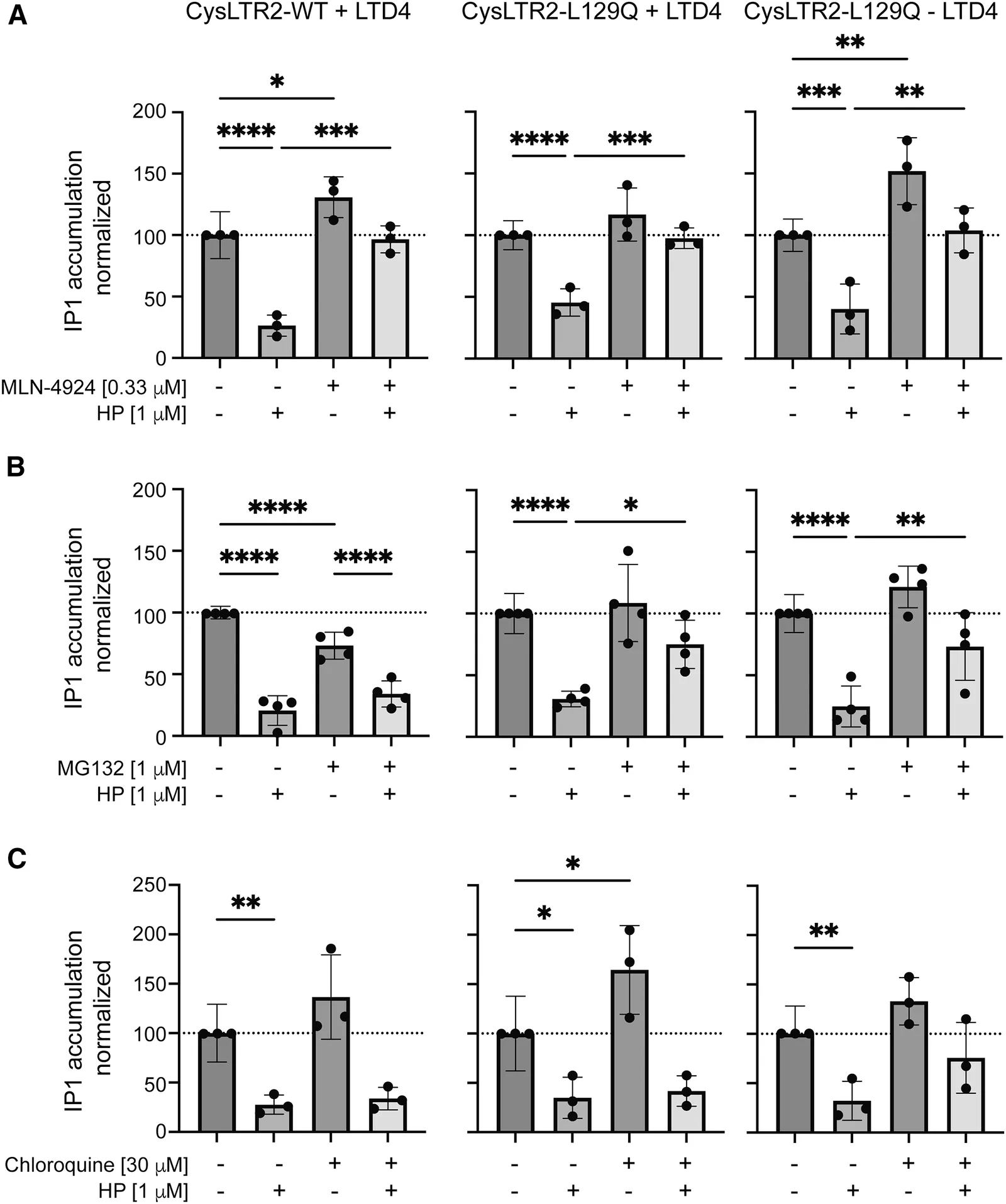
HaloPROTAC3 treatment causes a decrease in signaling due to activation of the ubiquitin-proteasome degradation pathway (A) IP_1_ accumulation after pretreatment with MLN-4924 and treatment with HaloPROTAC3 (HP). (B) IP_1_ accumulation after pretreatment with MG132 and treatment with HP. (C) IP_1_ accumulation after pretreatment with chloroquine and treatment with HP. Each data point represents the mean signal ±SD from three technical replicates. Conditions were compared using a multiple comparisons one-way ANOVA. Statistical significance is indicated as follows for all graphs: ns, *p* > 0.05; ∗, *p* ≤ 0.05; ∗∗, *p* ≤ 0.01; ∗∗∗, *p* ≤ 0.001; and ∗∗∗∗, *p* ≤ 0.0001.

We next tested MG132, a proteasomal inhibitor, which inhibits the 26S proteasome by targeting the 20S proteasome core, blocking catalytic activity (Figure 5B). The addition of MG132 caused a modest ∼20% decrease in IP_1_ accumulation in the case of CysLTR2-WT, however, there was no significant difference in the IP_1_ accumulation observed with CysLTR2-L129Q both in the presence and absence of LTD4. As observed earlier, HaloPROTAC3 treatment caused a ∼50–60% decrease in IP_1_ accumulation for both mutant and WT receptor constructs with and without agonist treatment. When HaloPROTAC3 was added in the presence of MG132 to CysLTR2-WT, we did not observe a significant increase in signaling. However, IP_1_ signaling mediated by CysLTR2-L129Q in both the presence and absence of LTD4 significantly increased when HaloPROTAC3 was added in the presence of MG132 as compared with addition of HaloPROTAC3 alone.

To further understand the mechanism of action, we used chloroquine, a lysosomotropic agent that increases the pH of the lysosome and therefore inhibits lysosomal degradation (Figure 5C). With the addition of chloroquine alone, we saw a significant increase in CysLTR2-L129Q signaling in the presence of LTD4. HaloPROTAC3 treatment alone showed a ∼50–60% decrease in IP_1_ accumulation for both mutant and WT receptor constructs. Treatment with HaloPROTAC3 in the presence of chloroquine showed no significant restoration of IP_1_ accumulation mediated by either CysLTR2-WT or CysLTR2-L129Q in the presence or absence of LTD4.

### CysLTR2-L129Q is degraded in primary human melanocytes

Since CysLTR2-L129Q is a driver for UM, we wanted to determine whether the HaloPROTAC3 system would show the same effects in a disease-relevant cell type. Therefore, we expressed the engineered CysLTR2-L129Q in primary melanocytes using a lentiviral stable transduction method. This method provided more stable receptor expression by integrating a stable gene into the host cell genome and allowed the receptor expression level to be titrated by adjusting the lentivirus MOI. Melanocytes were transduced with the CysLTR2-L129Q expression construct, and 12 h after transduction, various concentrations of HaloPROTAC3 and ent-HaloPROTAC3 were added for a 16-h incubation. Immunoblot analysis was conducted to visualize the protein abundance, again probing for the N-terminal OLLAS tag (Figure 6A). Using immunoblot analysis, we observed a dose-dependent decrease in the receptor protein abundance after the addition of HaloPROTAC3. At concentrations as low as 0.1 μM HaloPROTAC3, there was a ∼50% decrease in CysLTR2-L129Q protein abundance. At the highest concentration of HaloPROTAC3 (1 μM) there was a small but not statistically significant increase in protein abundance compared with treatment at 0.1 μM. The addition of the ent-HaloPROTAC3 resulted in a full restoration of receptor protein abundance.

**Figure 6:**
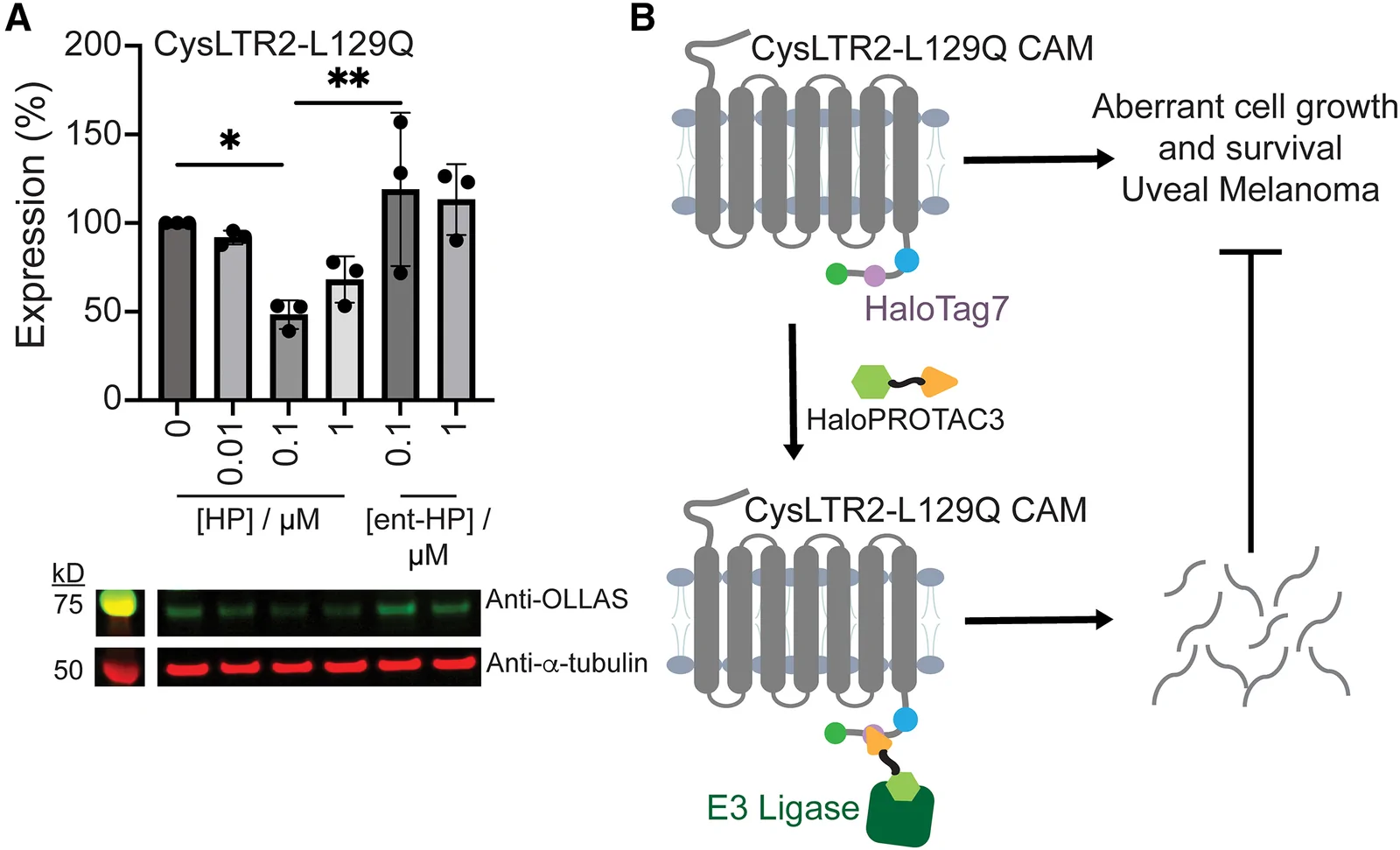
HaloPROTAC3 facilitates degradation of CysLTR2-L129Q CAM in primary melanocytes (A) CysLTR2-L129Q was expressed in primary human melanocytes using a lentiviral expression system. After treatment with HaloPROTAC3 (HP) or enantiomer HaloPROTAC3 (ent-HP), receptor protein levels were quantified using immunoblot analysis. The bar graph represents data from three independent experiments. One of the three immunoblot images is shown below the bar graph. Data were normalized using LiCOR Empiria Studio against a loading control and then normalized and compared to the condition without HP using a one-way ANOVA with multiple comparisons. Each data point represents the signal from one lane in one individual experiment. Statistical significance is indicated as follows: ns, *p* > 0.05; ∗, *p* ≤ 0.05; ∗∗, *p* ≤ 0.01; ∗∗∗, *p* ≤ 0.001; and ∗∗∗∗, *p* ≤ 0.0001. (B) Schematic summary of HaloPROTAC3-induced degradation of CysLTR2-L129Q CAM, which activates Gα_q_ signaling. HaloPROTAC3 binds to the HaloTag7 on the engineered receptor to cause recruitment of the VHL E3 ligase to promote the ubiquitination and degradation of the CAM.

## Discussion

Our overall aim was to determine the feasibility of using PROTACs to induce ubiquitin-proteasome-dependent degradation of GPCRs. We used a CDT, HaloTag7, and the corresponding degrader, HaloPROTAC3, to test the focused library of engineered GPCRs (Figure S2).^46^^,^^47^ When designing the expression constructs, we were concerned that transient transfection might lead to overexpression of the protein of interest (POI) so that the effect of the degrader would be masked. Therefore, we tested a weak promoter system to improve the dynamic range of the degrader assay. We first used a herpes simplex virus thymidine kinase (HSV-TK) weak promoter system in a transient transfection model. We compared the HSV-TK weak promoter construct against a previously characterized CMV promoter reference construct as a benchmark to understand the changes in signaling of the weaker HSV-TK promoter. Signaling and expression data show that the HSV-TK constructs express and function as expected (Figures 1C and 1D). Using a HiBiT quantification assay, a time-dependent and dose-dependent decrease in protein levels upon the addition of HaloPROTAC3 for the HSV-TK promoter was observed (Figures 1E and 1F). As expected, there was no dose-dependent decrease in protein levels with the strong CMV promoter constructs upon addition of HaloPROTAC3 (Figure S1). These results show that using the weaker promoter allows for the required dynamic range in the assay.

We constructed a focused library of engineered Gα_q_-coupled GPCRs with potential relevance to the molecular pathophysiology of cancer. Each CDT-containing GPCR was degraded after the addition of HaloPROTAC3, as judged by immunoblot analysis using a mAb against an N-terminal epitope tag (Figure 2). Additionally, all receptors showed a dose-dependent decrease in agonist-dependent Gα_q_ activation upon treatment with HaloPROTAC3. However, treatment with ent-HaloPROTAC3 did not result in GPCR degradation or decrease in agonist-dependent signaling. These results suggest that HaloPROTAC3 targets the expressed receptors and promotes the recruitment of the VHL E3 ligase, which then causes a reduction in protein expression and downstream signaling. These differences in efficacy of HaloPROTAC3 among the GPCRs tested could be due to a variety of factors, including the cellular location of the respective GPCRs, the accessibility of the cytoplasmic tail, the specific ubiquitination site, and the accessibility of HaloPROTAC3 to bind to the engineered HaloTag7. Specifically, GPCRs can be expressed both on the plasma membrane and intracellularly; therefore, the receptors that are expressed largely on the plasma membrane might degrade at a different rate than the receptors expressed internally. Additionally, GPCRs can be ubiquitinated on the intracellular C-terminal tail or one of the three intracellular loops; therefore, different ubiquitination locations could affect the efficiency of ubiquitination and, therefore, degradation.^26^ The accessibility of HaloPROTAC3 to bind specifically in the HiBiT quantification assay; the CDT-tag system could be limiting the accessibility of the HiBiT tag on the receptor construct for the LgBiT complementation, which could account for differences compared to the immunoblot. Therefore, receptor construct optimization is required for further CDT GPCR studies.^39^

We next focused on studying CysLTR2 and the CysLTR2-L129Q mutant, which is constitutively active and has been shown to be a driver oncoreceptor in UM. In earlier work, we could not identify a small molecule inverse agonist that could decrease the constitutive signaling of CysLTR2-L129Q.^21^ Using receptor constructs engineered with the CDT, we observed a decrease in receptor levels after addition of the HaloPROTAC3, while no effect was observed when the ent-HaloPROTAC3 was added to CysLTR2-L129Q (Figure 3A). Treatment with HaloPROTAC3 also decreased the downstream signaling of agonist-dependent and agonist-independent signaling by both CysLTR2 and the CysLTR2-L129Q mutant (Figure 3B). Although the IP_1_ accumulation assay is an indirect method for measuring the degradation of CysLTR2 WT and L129Q, the constitutive activity of CysLTR2-L129Q is the therapeutically relevant aspect; therefore, we proceeded to study the HaloPROTAC3 mechanism on the constitutive activity through the IP_1_ accumulation assays.

PROTACs function by forming a ternary complex between a POI and a targeted E3 ligase. The results of IP_1_ accumulation assays suggested that HaloPROTAC3 mediated the formation of a ternary complex between CysLTR2-L129Q and VHL E3 ligase. Pre-treatment with the VHL E3 ligase ligand, VL285, blocked the HaloPROTAC3-mediated decrease in CysLTR2-L129Q signaling (Figure 4A). Likewise, pre-treatment with the HaloTag7 ligand, 7-bromo-1-heptanol, also blocked the HaloPROTAC3-mediated decrease in CysLTR2-L129Q signaling (Figure 4B). Together, these experiments suggest that the HaloPROTAC3 must bind to both the receptor, through the HaloTag7, and the VHL E3 ligase to cause a decrease in receptor-mediated IP_1_ accumulation, which is consistent with the formation of a PROTAC-mediated ternary complex.

Results of experiments with the neddylation inhibitor MLN4924 suggest that the activation of Cullin-RING ligases and ubiquitination are a part of the downregulation or recycling of these GPCRs (Figure 5A). Pre-treatment of MLN4924 prevented the decrease in IP_1_ accumulation facilitated by HaloPROTAC3 treatment of CysLTR2-L129Q and CysLTR2-WT, presumably by inhibiting the activation of the ubiquitin-proteasome degradation pathway. In addition, pre-treatment with the proteasomal inhibitor MG132 prevented the effect of HaloPROTAC3 on CysLTR2-L129Q in the presence and absence of LTD4, suggesting the proteasomal degradation system is required to facilitate a decrease in receptor signaling (Figure 5B). Interestingly, the effect of MG132 pre-treatment on the CysLTR2-WT was less pronounced. Previous studies have shown that CysLTR2-L129Q is minimally expressed on the cellular surface as compared with CysLTR2-WT.^48^ The intracellular location does not seem to have an effect on the upstream activation of the ubiquitin-proteasome degradation pathway of CysLTR2-WT, as MLN4924 showed an increase in IP_1_ accumulation, which is unsurprising as GPCRs can be ubiquitinated regardless of cellular location.^26^ However, this difference in intracellular localization between the WT and CAM receptors could account for changes in proteasomal degradation. Ubiquitination of GPCRs can result in a variety of responses, including trafficking, recycling, or degradation; therefore, differences in receptor intracellular localization could result in higher proteasomal degradation of CysLTR2-L129Q compared with CysLTR2-WT. Future studies using a protein-specific PROTAC could further understand the effects of cellular location on proteasomal degradation. These experiments could include image-based assays, using lysosomal and proteasomal markers to visualize the receptor subcellular location and trafficking pathways.

Pre-treatment of chloroquine resulted in a significant increase in CysLTR2-L129Q mediated signaling in the presence of LTD4, suggesting that the lysosome plays a role in the internalization and degradation of the agonist ligand-activated mutant receptor. Treatment with HaloPROTAC3 in the presence of chloroquine showed no significant rescue of IP1 accumulation mediated by CysLTR2-WT or CysLTR2-L129Q in the presence or absence of LTD4, suggesting that the decrease in activity by HaloPROTAC3 is not reliant on lysosomal function alone. Overall, these results show that HaloPROTAC3 decreases the amount of functional CysLTR2-L129Q and suggest that the decrease in signaling is dependent on the ubiquitin-proteasomal degradation pathway. We hypothesize that in the case of the agonist-ligand activated CysLTR2-L129Q, treatment with HaloPROTAC3 favors the proteasomal degradation pathway as opposed to the lysosomal pathway (Figure 5C).

The effective degradation of CysLTR2-L129Q in melanocytes indicates that PROTAC technology can be employed therapeutically in UM. CysLTR2-L129Q constitutively activates the Gα_q_ signaling pathway to promote aberrant cell growth, which ultimately drives malignant transformation of melanocytes. Using primary human melanocytes and a lentiviral expression system, we demonstrated that treatment with HaloPROTAC3 but not the ent-HaloPROTAC3, decreases protein abundance again, suggesting that the recruitment of the VHL E3 ligase is required for the observed effect (Figure 6A). The slight rebound in protein abundance at the highest concentration of HaloPROTAC3 could be caused by the so-called “hook” effect. The “hook” effect describes a biphasic concentration dependence where higher concentrations of a scaffold, the PROTAC, suppress the interaction, a concept originally established by studying the MAP kinase activation cascades.^49^^,^^50^ In the case of a PROTAC, this suppression is due to PROTAC compounds saturating the binding sites of the receptor and E3 ligase separately and preventing ternary complex formation. Our data using the engineered CDT CysLTR2-L129Q show that HaloPROTAC3 causes recruitment of VHL E3 ligase and a decrease in protein expression that is consistent with a ubiquitin-proteasome-dependent degradation mechanism (Figure 6B).

In this study, we use the CDT system to test the potential feasibility of PROTAC-mediated degradation on a panel of GPCRs as a proof-of-concept study. However, the CDT system is an engineered tag system, and the introduction of the tag itself might result in changes in receptor functioning, subcellular location, and trafficking independent of HaloPROTAC3-mediated degradation. Additionally, the CDT system would not be transferable *in vivo*. Therefore, creation of individual PROTACs for selected therapeutically relevant GPCRs is ultimately required. Additionally, characterization of a specific PROTAC would need to include degradation efficacy and PROTAC effects on subcellular location and trafficking, downstream signaling, and phenotypic effects. However, the CDT system provides a screening approach that provides information on the suitability of exploring PROTAC technology for membrane proteins such as GPCRs. Additionally, HaloPROTAC3-mediated degradation shows high target selectivity, as the HaloTag7 has no endogenous binding partners. Broader proteome-wide effects include off-target effects of E3 ligase recruitment, including disrupting normal VHL E3 ligase activity, and utilization of the ubiquitin-proteasome system could result in altered turnover for endogenous proteins. Therefore, our study uses the HaloPROTAC3 CDT system as a proof-of-concept to evaluate the feasibility of degrading GPRCs with PROTAC technology, however specific anti-GPCR PROTACs would need extensive characterization.

Our work suggests that a specific CysLTR2-L129Q PROTAC could have therapeutic efficacy in melanoma. PROTACs targeting mutated proteins such as BRAF-V600E have already been shown to be successful in melanoma models, indicating that PROTACs are effective in melanoma and that an upstream CysLTR2-L129Q PROTAC could be an effective UM therapeutic.^51^ Additionally, PROTACs have been used to target constitutively active mutant proteins, such as JAK mutants and BRAF mutants, resulting in benefits to pathology.^52^^,^^53^ Future work to create such a PROTAC would need to consider the specific binding location of the PROTAC to CysLTR2-L129Q. The ideal GPCR PROTAC would bind to an intracellular allosteric binding site (IABS); however, the discovery and optimization of such allosteric compounds is challenging. We have previously proposed a model for a specific CysLTR2 PROTAC using a known antagonist.^36^ Additionally, we have previously described methodologies using bioorthogonal tethering to probe for novel intracellular allosteric or cryptic binding sites as starting points for the discovery of allosteric ligands.^36^^,^^54^^,^^55^ Molecular dynamics simulations using large chemical libraries could be used to discover potential intracellular binders and binding pockets. These potential intracellular binders could then be tested for binding using methods such as microscale thermophoresis (MST).^56^^,^^57^^,^^58^ As these binders do not need to affect the pharmacology, validating binding through MST would be sufficient for further PROTAC development. Previous research which determines favorable chemical conditions for the PROTAC linker would also provide insight into creating a CysLTR2-L129Q PROTAC.^59^ Altogether, it could be possible to create a specific CysLTR2-L129Q PROTAC using bioorthogonal tethering methods to determine optimal surfaces for ubiquitination, and large library molecular dynamics simulations to discover intracellular binders.

### Limitations of the study

A primary limitation of this study is the reliance on the engineered chemical degron tag (CDT) system. Because this approach requires the engineering of a tag to the receptor tail, the tag itself could alter basal receptor functioning, subcellular localization, or trafficking pathways independently of any small molecule-mediated degradation. Furthermore, these fused protein tags may artificially influence the ubiquitination kinetics of the target, potentially promoting ubiquitination on the tag rather than the protein of interest. Because the CDT system relies on an artificial fusion protein, it is not directly transferable *in vivo*, which limits the immediate physiological translatability of these specific constructs. Technical constraints inherent to our assay configurations also warrant consideration. First, steric accessibility or competitive binding dynamics of the degrader molecule could influence the readout of the bioluminescent peptide tag assay system. Second, while the inositol phosphate (IP1) accumulation assay is a robust and necessary method to quantify the constitutive signaling activity of the mutant receptor, it represents an indirect measure of physical protein degradation. Future studies utilizing endogenous, untagged receptor models will be valuable to validate these target degradation kinetics in more native biological environments.

## Resource availability

### Lead contact

Further information and requests for resources and reagents should be directed to and will be fulfilled by the lead contact, Thomas Sakmar (sakmar@rockefeller.edu).

### Materials availability

All plasmids generated in this study are available from the lead contact upon request with a completed materials transfer agreement.

### Data and code availability

• All data reported in this paper will be shared by the lead contact upon request.
• This paper does not report original code.
• Any additional information required to reanalyze the data reported in this paper is available from the lead contact upon request

## Acknowledgments

V.R.S., Y.C., and C.B. were supported by the Tri-Institutional PhD Program in Chemical Biology (NIH T32 GM136640-Tan). Y.C. is supported by a PhRMA Foundation Predoctoral Fellowship in Drug Discovery. We acknowledge grant support from the 10.13039/100013961Robertson Foundation and the Danica Foundation to T.P.S.

## Author contributions

Conceptualization, V.R.S., T.H., and T.P.S.; formal analysis, V.R.S., Y.C., and C.B.; investigation, V.R.S., Y.C., and C.B.; methodology, V.R.S., Y.C., T.H., and T.P.S.; project administration, V.R.S., T.H., and T.P.S.; writing – review and editing V.R.S., Y.C., C.B., T.H., and T.P.S.; funding acquisition, T.H., and T.P.S.; supervision, T.P.S.

## Declaration of interests

The authors declare no competing interests.

## Declaration of generative AI and AI-assisted technologies in the manuscript preparation process

The authors did not use AI in the preparation of this manuscript.

## STAR★Methods

### Key resources table

**Table:** 

REAGENT or RESOURCE	SOURCE	IDENTIFIER
**Antibodies**		

anti-OLLAS rat mAb	Park et al.^60^	N/A
anti-α-tubulin mouse mAb	Cell Signaling Technologies	Cat#3873S; RRID: AB_1904178
IRDye 680RD Goat anti-mouse IgG	LI-COR Biosciences	Cat#926–68070; RRID: AB_10956588
IRDye 800CW Goat anti-rat IgG	LI-COR Biosciences	Cat#926–32219; RRID: AB_1850025

**Bacterial and virus strains**		

pLV-eGFP	Addgene Enomoto et al.^61^	#36083
pMDLg/pRRE	Addgene	#12251
pRSV-Rev	Addgene	#12253
pMD2.G	Addgene	#12259

**Chemicals, peptides, and recombinant proteins**		

HaloPROTAC3	Promega	Cat# GA3110
VH285-PEG4-C4-Cl (HaloPROTAC3)	MedChem Express	HY-111997
Ent-HaloPROTAC3	Promega	Cat# GA4110
Lithium Chloride	Sigma Aldrich	Cat#L4408
Dulbecco’s Modified Eagle’s Medium (DMEM) GlutaMAX	ThermoFisher Scientific (Gibco)	Cat# 10566–016
FluoroBrite DMEM	ThermoFisher Scientific (Gibco)	Cat# A18967-01
Dulbecco’s phosphate-buffered saline without calcium and magnesium 10× (DPBS)	ThermoFisher Scientific (Gibco)	Cat# 14200–075
Poly-d-lysine hydrobromide	Sigma Aldrich	Cat# P6407
Fetal Bovine Serum (FBS)	Atlanta Biologicals, Inc.	Cat#S11150
0.25% Trypsin-EDTA 1X	ThermoFisher Scientific (Gibco)	Cat# 25200–056
L-Glutamine 200 mM 100x	ThermoFisher Scientific (Gibco)	Cat# 25030–081
Lipofectamine 2000 Transfection Reagent	ThermoFisher Scientific	Cat# 11668019
HEPES Buffer, 1M Solution, pH 7.3	Corning	Cat#25–060-CI
Bovine serum albumin (BSA) fraction V, fatty acid-free	Fisher Bioreagents	Cat# BP1605-100
DpnI	New England Biolabs	Cat# R0176S
Q5 Hot Start High-Fidelity DNA Polymerase	New England Biolabs	Cat# M0491L
Deoxynucleotide (dNTP) Solution Mix	New England Biolabs	Cat# N0447S
Luria-Bertani Broth, Miller	HIMEDIA	Cat#M1245
Dermal cell basal medium	ATCC	PCS-200–030
melanocyte growth kit	ATCC	PCS-200–41
Lenti-X Concentrator	Takara Bio	Cat#631231
polybrene	Sigma-Aldrich	TR-1003-G
LTD4	Cayman Chemical	Cat# 20310
Histamine	Tocris	Cat# 3545
BAM8-22	Abcam	Cat# ab120178
Nateglinide	Cayman Chemical	Cat# 23320
1-Oleoyl lysophosphatidic acid sodium salt	Tocris	Cat# 3854
3-[(1*R*)-1-Hydroxy-2-(methylamino)ethyl]phenol	Sigma Aldrich	Cat# CDS000481
Uridine 5′-diphosphate disodium salt hydrate	Sigma Aldrich	Cat# 94330
Carbamoylcholine chloride (Carbachol)	Abcam	Cat# ab141354-1g
MLN 4924	Tocris	Cat# 6499
7-bromo-1-heptanol	ThermoFisher Scientific	Cat# H54762.03
VL285	MedChemExpress	Cat# HY111663
MG132	Tocris	Cat# 1748
Chloroquine diphosphate salt	Sigma Aldric	Cat# C6628

**Critical commercial assays**		

IP-One Gq Kit	Cisbio	Cat# 62IPAPEC
Nano-Glo HiBiT Lytic Detection System	Promega	Cat# N3030
NEBuilder HiFi DNA Assembly Cloning Kit	New England Biolabs	Cat# E5520S
QuikChange Lightning Site-Directed Mutagenesis Kit	Agilent	Cat# 210519
DNA Clean and Concentrator-5	Zymo Research	Cat# D4014
QIAprep Spin Miniprep Kit	Qiagen	Cat# 27106
QIAGEN Plasmid Maxi Kit	Qiagen	Cat# 12162
ZymoPURE II Plasmid Midiprep Kit	Zymo Research	Cat#D4200
DC Protein Assay	BIO-RAD	Cat#5000111

**Experimental Models: Cell Lines**		

HEK293T Cells	ATCC	CRL-3216
*E. coli* strain DH5α	New England Biolabs	N/A
Primary Epidermal Melanocytes; Normal, Human, Neonatal (HEMn) (Male)	ATCC	PCS-200-012

**Oligonucleotides**		

See Tables S1–S3 for oligonucleotides used in these studies	This paper (purchased from IDT)	N/A

**Recombinant DNA**		

CysLTR2-GFP10-1D4 cDNA in pcDNA3.1(+)	Ceraudo et al.^16^	N/A
HSV-TK-SP-FLAG-OLLAS-CysLTR2-HiBiT-HT7-1D4	This paper (purchased from Azenta Life Sciences)	N/A
CMV-SP-FLAG-OLLAS-CysLTR2-HiBiT-HT7-1D4	This paper (purchased from Azenta Life Sciences)	N/A
HSV-TK-SP-FLAG-OLLAS-CysLTR1-HiBiT-HT7-1D4	This paper	N/A
HSV-TK-SP-FLAG-OLLAS-HRH1-HiBiT-HT7-1D4	This paper	N/A
HSV-TK-SP-FLAG-OLLAS-MRGPRX1-HiBiT-HT7-1D4	This paper	N/A
HSV-TK-SP-FLAG-OLLAS-MRGPRX4-HiBiT-HT7-1D4	This paper	N/A
HSV-TK-SP-FLAG-OLLAS-LPAR5-HiBiT-HT7-1D4	This paper	N/A
HSV-TK-SP-FLAG-OLLAS-ADRA1A-HiBiT-HT7-1D4	This paper	N/A
HSV-TK-SP-FLAG-OLLAS-P2RY2-HiBiT-HT7-1D4	This paper	N/A
HSV-TK-SP-FLAG-OLLAS- P2RY4-HiBiT-HT7-1D4	This paper	N/A
HSV-TK-SP-FLAG-OLLAS-CHRM1-HiBiT-HT7-1D4	This paper	N/A
HSV-TK-SP-FLAG-OLLAS-CysLTR2 L129Q-HiBiT-HT7-1D4	This paper	N/A
pLV- CMV-SP-FLAG-OLLAS-CysLTR2-HiBiT-HT7-1D4	This paper	N/A

**Software and algorithms**		

Gen5 Microplate Reader and Imager Software	BioTek	https://www.biotek.com
SoftMax Pro 6.4.2	Molecular Devices	https://www.moleculardevices.com
Prism 10 (Version 10.4.1)	GraphPad Software	https://www.graphpad.com
Empiria Studio Software	LicorBio	https://www.licorbio.com/empiria-studio

**Other**		

384 well, with lid, low volume, tissue culture treated black with clear bottom, polystyrene, non-pyrogenic	Corning	Cat#3542
96 Well, F-bottom/chimney well, black, μclear, advanced TC, sterile, with lid	Greiner Bio-One	Cat# 655 986

### Experimental model and study participant details

#### Plasmids

Two custom plasmids were purchased from Azenta Life Sciences encoding SP-FLAG-OLLAS-CysLTR2-SmBiT-HT7-1D4 with either a cytomegalovirus (CMV) promoter or a herpes simplex virus thymidine kinase (HSV-TK) promoter. Vector abbreviations are as follows: SP, signal peptide; FLAG, FLAG epitope tag; OLLAS, OLLAS epitope tag; CysLTR2, cysteinyl leukotriene receptor 2; HiBiT, small nanoluciferase complementary peptide; HT7, HaloTag7; 1D4, 1D4 epitope tag.

NEBuilder HiFi DNA Assembly Tool was used to assemble all Gα_q_-couple screening plasmids. These plasmids were made using both the HSV-TK promoter construct (SP-FLAG-OLLAS-CysLTR2-SmBiT-HT7-1D4) and specific receptor constructs from the codon-optimized DuET library created from the PRESTO-Tango Library purchased from the Illuminating the Druggable Genome NIH Program.^44^ Reactions were done in two parts, starting with a polymerase chain reaction (PCR) using Q5 Hot Start High-Fidelity DNA Polymerase (New England Biolabs) to amplify the HSV-TK promoter construct as the backbone and each DuET library receptor construct as an insert. Primers for amplification were created using the NEBuilder Assembly Tool on the NEB website. The PCR reactions were carried out following the manufacturer’s recommendations. Subsequently, 1 μL of DpnI and 2.5 μL of 10X CutSmart Buffer were added and the mixture which was incubated at 37 °C for 30 min. The PCR products were then cleaned and concentrated using the DNA Clean & Concentrator-5 kit (Zymo Research). The resulting PCR product’s concentration was quantified using a NanoDrop. Final construct assembly was performed for 15 min at 50 °C using the NEBuilder HiFi DNA Assembly kit in 20 μL total reaction volume using 0.033 pmol vector and 0.066 pmol insert. Following the reaction incubation, 1 μL of assembled product was used to transform NEB 5-α Competent *Escherichia coli* cells. Cells were then spread on Luria-Bertani (LB)-agar plates containing 100 μg/mL ampicillin (Amp). Colonies were picked and Sanger sequencing (Azenta Life Sciences) confirmed final DNA constructs.

QuikChange Lightning Site-Directed Mutagenesis Kit and the HSV-TK CysLTR2 construct were used to create the CysLTR2-L129Q mutant. Primers were designed using the QuikChange Primer Design web tool. PCR reactions were conducted based on the manufacturer’s instructions with the modification of using half-volume reactions and using 50 ng of HSV-TK CysLTR2 template. Following the reaction, 1 μL of PCR product was used to transform XL10-Gold ultracompetent cells. Cells were spread on LB-agar plates containing 100 μg/mL Amp, colonies were picked, and DNA was confirmed as described above.

Similarly, the NEBuilder HiFi DNA Assembly protocol was used to create the viral pLV-CysLTR2-L129Q construct. The plasmid was created using the CVM promoter construct (SP-FLAG-OLLAS-CysLTR2-SmBiT-HT7-1D4) and a pLV-eGFP construct bought from Addgene (#36083).^61^

#### Cell culture and transfection

HEK293T cells (America Type Culture Collection, #CRL-32166) with passage number of 6–25 were maintained in Dulbecco’s Modified Eagle Medium containing 4.5 g/L D-Glucose (DMEM-GlutaMax, Thermo Fisher Scientific) supplemented with 15 mM *N*-2-hydroxyethylpiperazine-*N′*-2-ethanesulphonic acid (HEPES, Corning) and 10% (v/v) Fetal Bovine Serum Premium (FBS, Atlanta Biologicals, Inc.).

Primary epidermal melanocytes (male) (American Type Culture Collection, #PCS-200–012) were thawed at passage number two and maintained in Dermal cell basal medium (American Type Culture Collection, #PCS-200–030) supplemented with a melanocyte growth kit (American Type Culture Collection #PCS-200–41) in T25 flasks and kept at 37 °C, 5% CO_2_. Cell lines were tested negative for mycoplasma contamination.

### Method details

#### HEK293T transfection

A 96-well microtiter plate (polystyrene, black-walled, chimney, flat bottom, Greiner Bio-One) or a 384-well microtiter plate (polystyrene, black-walled, clear flat bottom, Corning) were prepared using UV/ozone treatment for 8 min and then treated with 0.1 mg/mL poly-d-lysine (PDL, Sigma-Aldrich) for 1 h at room temperature (RT). Cells were then transfected with 2 pg of total DNA per cell (1:1 ratio of receptor to mock DNA). The DNA was diluted in FluoroBrite-DMEM (Thermo Fisher Scientific). Lipofectamine 2000 (2.5 μL/μg DNA) (Thermo Fisher Scientific) was diluted in FluoroBrite-DMEM, mixed by inversion, and incubated at RT for 5 min. Equal volumes of lipofectamine mixture were added to individual diluted DNA, which were then mixed and incubated at RT for 20 min. Subsequently, HEK293T cells were dissociated from a 10-cm culture plate using Trypsin-EDTA (0.25%), phenol red (Thermo Fisher Scientific) and resuspended to 20,000 cells per 20 μL for 96-well plates or 7,000 cells per 5 μL for 384-well plates in FluoroBrite-DMEM supplemented with 30 mM HEPES, 8 mM L-glutamine (Thermo Fisher Scientific), and 20% fetal bovine serum (Atlanta Biologicals). For each individual experiment, all cells were taken from initial plate or pooled plates before seeding into 96-well or 384-well plate. When the 20-min incubation was complete, an equal volume of suspended cells was added to the DNA and lipofectamine mixture. This mixture was then seeded into the designated wells (40 μL for the 96-well plate or 10 μL for the 384-well plate) of the assay plate. Assay plates were then placed in an incubator at 37 °C, 5% CO_2_ for 12 to 24 h before HaloPROTAC3 addition and expression or signaling assays.

For immunoblot assays, cells were similarly transfected with 2 pg of total DNA per cell (1:1 ratio of receptor to mock DNA). Cells were transfected in the same manner as the previous assay using Lipofectamine 2000 and seeded directly into 6-well tissue culture plates. For immunoblot assays, cells were resuspended to 700,000 per well for GPCR panel experiments and 1,400,000 cells per well for CysLTR2-L129Q experiments in FluoroBrite-DMEM supplemented with 30 mM HEPES, 8 mM L-glutamine, and 20% fetal bovine serum. For 6-well tissue culture plates, 2 mL of the DNA/Lipofectamine 2000/cell mixture was seeded into the designated well.

#### Lentiviral production

Lentiviral particles were generated in HEK293T cells using a third-generation packaging system. HEK293T cells were seeded in a 10-cm dish at 4,000,000 cells/dish. The next day, the HEK293T cells were co-transfected with 20 μg of total DNA per plate with the viral pLV-CysLTR2-L129Q construct or pLV-eGFP construct and packaging constructs including pMDLg/pRRE (Addgene #12251), pRSV-Rev (Addgene #12253), and pMD2.G (Addgene #12259) at a 3:1:1:1 ratio and diluted in 500 μL Opti-MEM (Gibco). Separately Lipofectamine 2000 (2.5 μL/μg DNA) (Thermo Fisher Scientific) was diluted in Opti-MEM, mixed by inversion, and incubated at RT for 5 min. Equal volumes of lipofectamine mixture were added to individual diluted DNA, which were then mixed and incubated at RT for 20 min. 1 mL DNA/lipofectamine mixture was then dropwise added to individual 10-cm dish plates. 12–18 h after transfection, the media were exchanged for Dulbecco’s Modified Eagle Medium containing 4.5 g/L D-Glucose (DMEM-GlutaMax, Thermo Fisher Scientific) supplemented with 15 mM *N*-2-hydroxyethylpiperazine-*N′*-2-ethanesulphonic acid (HEPES, Corning) and 10% (v/v) Fetal Bovine Serum Premium (FBS, Atlanta Biologicals, Inc.). Three days post-transfection, the supernatant containing virus was collected and filtered through a 0.45 μm syringe filter and then stored overnight at 4°C. Fresh DMEM media was added to the cells and then placed in a 37 °C 5% CO_2_ incubator overnight. The next day, the supernatant was collected, filtered, and combined with the previous supernatant containing the virus. Three volumes of filtered supernatant were then combined with one volume of Lenti-X Concentrator (Takara Bio Inc.) mixed, and then incubated overnight at 4 °C. The next day, the mixture was spun down, and the supernatant was discarded, and the resulting virus pellet was resuspended in 2 mL 1X PBS and stored at −80 °C until use. We determined the viral titer of the packaged CysLTR2-L129Q virus was 2.91 × 10^7^ TU/mL using the Lenti-X qRT-PCR Titration Kit (Takara #631235).

#### Lentiviral transduction

One day prior to transduction, melanocytes at passage three were reseeded in a 6-well plate at 20,000 cells per well. For transduction, the virus was thawed on ice before adding to complete media at a 1:20 concentration. 12 h after transduction, the media was exchanged for 1 mL of complete media containing 10 nM, 100 nM, or 1 μM HaloPROTAC3 or 1 μM ent-HaloPROTAC3 and placed in the incubator at 37 °C, 5% CO_2_ for 16 h.

#### Compound and HaloPROTAC3 addition

After 12 to 24 h of protein expression, cells were treated with HaloPROTAC3 (Promega) or enantiomer HaloPROTAC3 (Promega) and placed in the incubator at 37 °C, 5% CO_2_ for 16 h. For time course HiBiT experiment, HaloPROTAC3 was incubated for the designated time. HaloPROTAC3 was treated for 16 h in all assays other than the time course assay.

For the CysLTR2-L129Q inhibitor and competitor experiments, after 10 h of protein expression, cells were pretreated with inhibitors (MG132, Chloroquine, MLN4924), competitors (VL285, 7-bromo-1-heptanol) or vehicle in FluoroBrite-DMEM supplemented with 15 mM HEPES, 4 mM L-glutamine, and 10% fetal bovine serum. Cells were then placed back in the incubator at 37 °C, 5% CO_2_ for 2 h. Following inhibitor or competitor incubation, cells were treated with HaloPROTAC3 or enantiomer HaloPROTAC3. The assay plate was then placed in the incubator at 37 °C, 5% CO_2_ for 16 h.

For the immunoblot assays, after 24 h of expression for GPCR panel receptors or 12 h of CysLTR2-L129Q expression, cells were treated with 500 μL HaloPROTAC3 or enantiomer HaloPROTAC3 or vehicle at a 5x concertation. The plate was then placed in the incubator at 37 °C, 5% CO_2_ for 16 h.

#### HiBiT protein quantification assay

Following incubation with compound, cells were removed from the incubator and allowed to equilibrate to room temperature. In parallel, following the manufacturer’s instructions the LgBiT Protein was diluted 1:100, and the Nano-Glo HiBiT Lytic Substrate was diluted 1:50 in the Nano-Glo HiBiT Lytic Buffer (Promega). An equal volume of Lytic Buffer mixture to the cell volume was added to each well. The plate was then mixed for 3 min at 600 rpm before all luminescence was read on a SpectraMax i3x multi-mode microplate reader (Molecular Devices). The total luminescence was read every 5 min over 45 min.

#### IP-one accumulation assay

Following protein expression and HaloPROTAC3 incubation if required, cells were treated with 2 μL/well of agonist or vehicle, diluted in a pre-warmed stimulation assay buffer provided by the manufacturer (10 mM HEPES, 1 mM CaCl2, 0.5 mM MgCl2, 4.2 mM KCl, 146 mM NaCl, 5.5 mM glucose, pH 7.4) and supplemented with 0.1% (w/v) BSA and 50 mM lithium chloride, for 2 h at 37 °C, 5% CO_2_. Following stimulation, cells were lysed with the addition of 3 μL/well of d2-labeled d-myo-inositol-1-phosphate (IP_1_) analog and 3 μL/well of terbium cryptate-labeled anti-IP_1_ mAb in the lysis and detection buffer provided by the manufacturer. Plates were then mixed for 30 s at 1200 rpm and placed in the dark at RT to incubate for 1 h and 30 min to 2 h. The BioTek Synergy *NEO*2-TRF Hybrid multi-mode reader was used to readout time-resolved fluorescence signals. Plates were subjected to laser excitation at 337 nm; the fluorescence was then measured at 620 nm and 665 nm simultaneously.

#### Immunoblotting assay

Following HaloPROTAC3 incubation, HEK293T immunoblotting samples were washed with DPBS. After DPBS was aspirated, cells were dissociated with 1 mL DPBS supplemented with cOmplete, EDTA-free protease inhibitor cocktail tablet (PI, Roche). Cells were then transferred to a clean tube and pelleted at 500 G for 15 min, 4 °C. The supernatant was aspirated, and cell pellets were lysed in lysis buffer (50 mM Tris-HCl, pH 6.8, 100 mM NaCl, 1 mM CaCl2, 1% DDM, 0.1 mM PMSF, 0.12 mg (0.552 units) aprotinin, cOmplete Mini, EDTA-free protease inhibitor cocktail) for 1 h at 4 °C while nutating. After lysis, cell debris was then pelleted at 21000 G for 30 min, 4 °C. Lysates were then transferred to a clean tube and the total protein was normalized using a DC protein assay kit (Bio-Rad, Cat. #5000116) according to manufacturer’s instructions. Normalized protein samples were incubated with NuPAGE LDS Sample Buffer 4X and 100 mM DTT as preparation for gel electrophoresis and then frozen.

Melanocyte immunoblot samples were dissociated using 200 μL of EDTA Trypsin for 1 min. Following cell dissociation, 200 μL of neutralizing buffer is added to each well. Cells were collected and transferred to a clean tube and pelleted at 500 G for 15 min, 4 °C. The supernatant was aspirated, and cell pellets were washed with 1 mL DPBS supplemented with cOmplete, EDTA-free protease inhibitor cocktail tablet (Roche) and pelleted at 500 G for 5 min, 4 °C. Again, the supernatant was aspirated, and cell pellets were lysed using lysis buffer (50 mM Tris-HCl, pH 6.8, 100 mM NaCl, 1 mM CaCl2, 1% DDM, 0.1 mM PMSF, 0.12 mg (0.552 units) aprotinin, cOmplete, EDTA-free protease inhibitor cocktail). Total protein was normalized using the DC protein assay kit and normalized samples were then prepared in the same manner as the HEK293T immunoblot samples.

Samples were thawed before gel electrophoresis using a NuPAGE 4–12% Bis-Tris gel,1.0 mm x 17 well Mini Protein Gel in NuPAGE MES-SDS Running Buffer at a constant voltage of ∼120 V. Gel was transferred to Immobilon-FL PVDF membrane for immunoblot analysis. Blocking step was carried out using Intercept Blocking Buffer (IBB), before the primary antibody solution (IBB +0.1% Tween 20 + 1:2000 dilution anti-OLLAS rat monoclonal antibody (mAb) + 1:200 dilution anti-α-tubulin mouse mAb). Membranes were briefly washed 5 times in DPBS +0.1% Tween 20 before secondary antibody incubation (IBB +0.1% Tween 20 + 1:10,000 IRDye 800CW Goat anti-rat IgG +1:10,000 IRDye 680RD Goat anti-mouse IgG). All incubations were for 1 h at RT unless noted otherwise. Membranes were washed before imaging on LI-COR laser scanning imagers.

### Quantification and statistical analysis

#### HiBiT protein quantification assay

The three consecutive peak signal time points were then determined using wells transfected with mock DNA and not treated receptor. The luminescence signal was averaged over the predetermined three reads for each of the wells. The average signal of the three wells transfected with mock DNA (puc19) alone was then subtracted from each well as a background signal. The signal was then normalized to receptor no treatment or receptor treated with HaloPROTAC3 as the maximum. Three replicates of the normalized luminescence signal were then averaged for each plate. Normalized data were then compared to the condition without treatment using a multiple comparisons one-way ANOVA. See supplement for additional information. For all HiBiT Quantification Assay in Figure 1, statistical significance is indicated as follows for all graphs: ns, *p* > 0.05; ∗, *p* ≤ 0.05; ∗∗, *p* ≤ 0.01; ∗∗∗, *p* ≤ 0.001; and ∗∗∗∗, *p* ≤ 0.0001.

#### IP-one accumulation assay

Data were normalized to receptor with highest treatment of agonist or receptor without HaloPROTAC3 or enantiomer HaloPROTAC3 treatment. The mock without treatment was used as the minimum. For experiment comparing promoters, CMV vs. HSV-TK, data were normalized to CysLTR2-GFP10 with highest treatment of agonist. For all HaloPROTAC3 assays, the data were fit to a three-parameter inhibitor vs. response model, while experiments without HaloPROTAC3 were fit to a three-parameter agonist vs. response model. Data were plotted and fit to a sigmoidal response or horizontal line model and compared using Akaike’s Information Criterion. For all IP-One assay data in Figure 1, each point represents the mean ± S.D. of normalized data from three individual experiments. Statistical significance is indicated as follows for all graphs: ns, *p* > 0.05; ∗, *p* ≤ 0.05; ∗∗, *p* ≤ 0.01; ∗∗∗, *p* ≤ 0.001; and ∗∗∗∗, *p* ≤ 0.0001. See supplement for additional information.

#### Immunoblotting assay

LI-COR Empiria Studio 2.3 was used to quantitate the total raw signal from immunoblot. The lane normalization factor was calculated based on the internal loading control (α-tubulin) band for each lane using the equation:

$$Lane\;normalization\;factor=\frac{internal\;loading\;control\;signal\;per\;lane}{highest\;internal\;loading\;control\;signal}$$

The normalized signal was then calculated using the equation:

$$Normalized\;signal=\frac{Target\;band\;signal}{Lane\;normalization\;factor}$$

Finally, the signal was converted into a percentage of protein abundance by dividing each signal by each receptor without treatment. Data points represent one individual experiment, and the immunoblot image is a representative image of three independent experiments. Data were compared to the condition without HaloPROTAC3 using multiple comparisons one-way ANOVA. Statistical significance is indicated as follows for all graphs: ns, *p* > 0.05; ∗, *p* ≤ 0.05; ∗∗, *p* ≤ 0.01; ∗∗∗, *p* ≤ 0.001; and ∗∗∗∗, *p* ≤ 0.0001. See supplement for additional information.

## References

[1] Chaudhary P.K., Kim S. (2021). An Insight into GPCR and G-Proteins as Cancer Drivers. Cells.

[2] Feng C., Ooms J.F., Danen E.H.J., Heitman L.H. (2025). GPCR-G protein signalling and its mutational landscape in cancer-Driver or passenger. Br. J. Pharmacol..

[3] Dorsam R.T., Gutkind J.S. (2007). G-protein-coupled receptors and cancer. Nat. Rev. Cancer.

[4] Even-Ram S., Uziely B., Cohen P., Grisaru-Granovsky S., Maoz M., Ginzburg Y., Reich R., Vlodavsky I., Bar-Shavit R. (1998). Thrombin receptor overexpression in malignant and physiological invasion processes. Nat. Med..

[5] Rhodes D.R., Ateeq B., Cao Q., Tomlins S.A., Mehra R., Laxman B., Kalyana-Sundaram S., Lonigro R.J., Helgeson B.E., Bhojani M.S. (2009). AGTR1 overexpression defines a subset of breast cancer and confers sensitivity to losartan, an AGTR1 antagonist. Proc. Natl. Acad. Sci. USA.

[6] Filardo E.J., Graeber C.T., Quinn J.A., Resnick M.B., Giri D., DeLellis R.A., Steinhoff M.M., Sabo E. (2006). Distribution of GPR30, a seven membrane-spanning estrogen receptor, in primary breast cancer and its association with clinicopathologic determinants of tumor progression. Clin. Cancer Res..

[7] Müller A., Homey B., Soto H., Ge N., Catron D., Buchanan M.E., McClanahan T., Murphy E., Yuan W., Wagner S.N. (2001). Involvement of chemokine receptors in breast cancer metastasis. Nature.

[8] Taub J.S., Guo R., Leeb-Lundberg L.M.F., Madden J.F., Daaka Y. (2003). Bradykinin receptor subtype 1 expression and function in prostate cancer. Cancer Res..

[9] Hoshino K., Ishiguro H., Teranishi J.I., Yoshida S.I., Umemura S., Kubota Y., Uemura H. (2011). Regulation of androgen receptor expression through angiotensin II type 1 receptor in prostate cancer cells. Prostate.

[10] Reifenberger J., Wolter M., Weber R.G., Megahed M., Ruzicka T., Lichter P., Reifenberger G. (1998). Missense mutations in SMOH in sporadic basal cell carcinomas of the skin and primitive neuroectodermal tumors of the central nervous system. Cancer Res..

[11] Wang C., Wu H., Evron T., Vardy E., Han G.W., Huang X.P., Hufeisen S.J., Mangano T.J., Urban D.J., Katritch V. (2014). Structural basis for Smoothened receptor modulation and chemoresistance to anticancer drugs. Nat. Commun..

[12] Wu V., Yeerna H., Nohata N., Chiou J., Harismendy O., Raimondi F., Inoue A., Russell R.B., Tamayo P., Gutkind J.S. (2019). Illuminating the Onco-GPCRome: Novel G protein-coupled receptor-driven oncocrine networks and targets for cancer immunotherapy. J. Biol. Chem..

[13] Huber T., Horioka-Duplix M., Chen Y., Saca V.R., Ceraudo E., Chen Y., Sakmar T.P. (2024). The role of signaling pathways mediated by the GPCRs CysLTR1/2 in melanocyte proliferation and senescence. Sci. Signal..

[14] Van Raamsdonk C.D., Griewank K.G., Crosby M.B., Garrido M.C., Vemula S., Wiesner T., Obenauf A.C., Wackernagel W., Green G., Bouvier N. (2010). Mutations in GNA11 in uveal melanoma. N. Engl. J. Med..

[15] Moore A.R., Ceraudo E., Sher J.J., Guan Y., Shoushtari A.N., Chang M.T., Zhang J.Q., Walczak E.G., Kazmi M.A., Taylor B.S. (2016). Recurrent activating mutations of G-protein-coupled receptor CYSLTR2 in uveal melanoma. Nat. Genet..

[16] Ceraudo E., Horioka M., Mattheisen J.M., Hitchman T.D., Moore A.R., Kazmi M.A., Chi P., Chen Y., Sakmar T.P., Huber T. (2021). Direct evidence that the GPCR CysLTR2 mutant causative of uveal melanoma is constitutively active with highly biased signaling. J. Biol. Chem..

[17] Gusach A., Luginina A., Marin E., Brouillette R.L., Besserer-Offroy É., Longpré J.M., Ishchenko A., Popov P., Patel N., Fujimoto T. (2019). Structural basis of ligand selectivity and disease mutations in cysteinyl leukotriene receptors. Nat. Commun..

[18] Hauser A.S., Attwood M.M., Rask-Andersen M., Schiöth H.B., Gloriam D.E. (2017). Trends in GPCR drug discovery: new agents, targets and indications. Nat. Rev. Drug Discov..

[19] Yang D., Zhou Q., Labroska V., Qin S., Darbalaei S., Wu Y., Yuliantie E., Xie L., Tao H., Cheng J. (2021). G protein-coupled receptors: structure- and function-based drug discovery. Signal Transduct. Target. Ther..

[20] Usman S., Khawer M., Rafique S., Naz Z., Saleem K. (2020). The current status of anti-GPCR drugs against different cancers. J. Pharm. Anal..

[21] Ceraudo E., Horioka M., Mattheisen J.M., Hitchman T.D., Moore A.R., Kazmi M.A., Chi P., Chen Y., Sakmar T.P., Huber T. (2019). Uveal Melanoma Oncogene <em>CYSLTR2</em> Encodes a Constitutively Active GPCR Highly Biased Toward Gq Signaling. bioRxiv.

[22] Zhao L., Zhao J., Zhong K., Tong A., Jia D. (2022). Targeted protein degradation: mechanisms, strategies and application. Signal Transduct. Target. Ther..

[23] Krone M.W., Crews C.M. (2025). Next steps for targeted protein degradation. Cell Chem. Biol..

[24] Sakamoto K.M., Kim K.B., Kumagai A., Mercurio F., Crews C.M., Deshaies R.J. (2001). Protacs: chimeric molecules that target proteins to the Skp1-Cullin-F box complex for ubiquitination and degradation. Proc. Natl. Acad. Sci. USA.

[25] Schneekloth A.R., Pucheault M., Tae H.S., Crews C.M. (2008). Targeted intracellular protein degradation induced by a small molecule: En route to chemical proteomics. Bioorg. Med. Chem. Lett..

[26] Skieterska K., Rondou P., Van Craenenbroeck K. (2017). Regulation of G Protein-Coupled Receptors by Ubiquitination. Int. J. Mol. Sci..

[27] Tang X., Bian J., Li Z. (2022). Posttranslational modifications in GPCR internalization. Am. J. Physiol. Cell Physiol..

[28] Henne W.M., Buchkovich N.J., Emr S.D. (2011). The ESCRT pathway. Dev. Cell.

[29] Peeler J.C., Schedin-Weiss S., Soula M., Kazmi M.A., Sakmar T.P. (2017). Isopeptide and ester bond ubiquitination both regulate degradation of the human dopamine receptor 4. J. Biol. Chem..

[30] Moriyoshi K., Iijima K., Fujii H., Ito H., Cho Y., Nakanishi S. (2004). Seven in absentia homolog 1A mediates ubiquitination and degradation of group 1 metabotropic glutamate receptors. Proc. Natl. Acad. Sci. USA.

[31] Chaturvedi K., Bandari P., Chinen N., Howells R.D. (2001). Proteasome involvement in agonist-induced down-regulation of mu and delta opioid receptors. J. Biol. Chem..

[32] Cook L.B., Zhu C.C., Hinkle P.M. (2003). Thyrotropin-releasing hormone receptor processing: role of ubiquitination and proteasomal degradation. Mol. Endocrinol..

[33] Huber M.E., Toy L., Schmidt M.F., Vogt H., Budzinski J., Wiefhoff M.F.J., Merten N., Kostenis E., Weikert D., Schiedel M. (2022). A Chemical Biology Toolbox Targeting the Intracellular Binding Site of CCR9: Fluorescent Ligands, New Drug Leads and PROTACs. Angew. Chem. Int. Ed. Engl..

[34] Li Z., Lin Y., Song H., Qin X., Yu Z., Zhang Z., Dong G., Li X., Shi X., Du L. (2020). First small-molecule PROTACs for G protein-coupled receptors: inducing alpha (1A)-adrenergic receptor degradation. Acta Pharm. Sin. B.

[35] Lu A.S., Rouhimoghadam M., Arnatt C.K., Filardo E.J., Salem A.K. (2021). Proteolytic Targeting Chimeras with Specificity for Plasma Membrane and Intracellular Estrogen Receptors. Mol. Pharm..

[36] Saca V.R., Huber T., Sakmar T.P. (2025). G protein-coupled receptor-targeted proteolysis-targeting chimeras in cancer therapeutics. Mol. Pharmacol..

[37] Keen A.C., Jörg M., Halls M.L. (2024). The application of targeted protein degradation technologies to G protein-coupled receptors. Br. J. Pharmacol..

[38] Bensimon A., Pizzagalli M.D., Kartnig F., Dvorak V., Essletzbichler P., Winter G.E., Superti-Furga G. (2020). Targeted Degradation of SLC Transporters Reveals Amenability of Multi-Pass Transmembrane Proteins to Ligand-Induced Proteolysis. Cell Chem. Biol..

[39] Ortiz Zacarías N.V., Röth S., Broekhuis J.D., van der Es D., Moreau K., Heitman L.H. (2024). Inducing Receptor Degradation as a Novel Approach to Target CC Chemokine Receptor 2 (CCR2). Int. J. Mol. Sci..

[40] Essa K., Ortiz Zacarías N.V., Roth S., Xin B.T., de Winter S., van der Horst C., Bleijs B., de Heiden R.A., Voulgaraki R., Neubert E. (2025). Leveraging Targeted Protein Degradation for G Protein-Coupled Receptors: The Development of CCR2 Molecular Degraders. J. Med. Chem..

[41] Dixon A.S., Schwinn M.K., Hall M.P., Zimmerman K., Otto P., Lubben T.H., Butler B.L., Binkowski B.F., Machleidt T., Kirkland T.A. (2016). NanoLuc Complementation Reporter Optimized for Accurate Measurement of Protein Interactions in Cells. ACS Chem. Biol..

[42] Hall M.P., Unch J., Binkowski B.F., Valley M.P., Butler B.L., Wood M.G., Otto P., Zimmerman K., Vidugiris G., Machleidt T. (2012). Engineered luciferase reporter from a deep sea shrimp utilizing a novel imidazopyrazinone substrate. ACS Chem. Biol..

[43] Ali R., Ramadurai S., Barry F., Nasheuer H.P. (2018). Optimizing fluorescent protein expression for quantitative fluorescence microscopy and spectroscopy using herpes simplex thymidine kinase promoter sequences. FEBS Open Bio.

[44] Dahl L., Kotliar I.B., Bendes A., Dodig-Crnković T., Fromm S., Elofsson A., Uhlén M., Sakmar T.P., Schwenk J.M. (2023). Multiplexed selectivity screening of anti-GPCR antibodies. Sci. Adv..

[45] Merrill R.A., Song J., Kephart R.A., Klomp A.J., Noack C.E., Strack S. (2019). A robust and economical pulse-chase protocol to measure the turnover of HaloTag fusion proteins. J. Biol. Chem..

[46] Buckley D.L., Raina K., Darricarrere N., Hines J., Gustafson J.L., Smith I.E., Miah A.H., Harling J.D., Crews C.M. (2015). HaloPROTACS: Use of Small Molecule PROTACs to Induce Degradation of HaloTag Fusion Proteins. ACS Chem. Biol..

[47] Los G.V., Encell L.P., McDougall M.G., Hartzell D.D., Karassina N., Zimprich C., Wood M.G., Learish R., Ohana R.F., Urh M. (2008). HaloTag: a novel protein labeling technology for cell imaging and protein analysis. ACS Chem. Biol..

[48] Mattheisen J.M., Rasmussen V.A., Ceraudo E., Kolodzinski A., Horioka-Duplix M., Sakmar T.P., Huber T. (2024). Application of bioluminescence resonance energy transfer to quantitate cell-surface expression of membrane proteins. Anal. Biochem..

[49] Dickens M., Rogers J.S., Cavanagh J., Raitano A., Xia Z., Halpern J.R., Greenberg M.E., Sawyers C.L., Davis R.J. (1997). A cytoplasmic inhibitor of the JNK signal transduction pathway. Science.

[50] Chapman S.A., Asthagiri A.R. (2009). Quantitative effect of scaffold abundance on signal propagation. Mol. Syst. Biol..

[51] Alhassan S.O., Abd Elmageed Z.Y., Errami Y., Wang G., Abi-Rached J.A., Kandil E., Zerfaoui M. (2025). BRAF(V600E)-PROTAC versus inhibitors in melanoma cells: Deep transcriptomic characterization. Clin. Transl. Med..

[52] Wang C., Chen Z., Wang S., Wang J., Yu P., Huang C., Wu Y., Zhao Z., Mei W., Li H., Zhu L. (2025). Discovery of a Proteolysis-Targeting Chimera Degrader of JAK2 as a Potential Therapeutic Agent for JAK2-Mediated Myeloproliferative Neoplasms. J. Med. Chem..

[53] Alabi S., Jaime-Figueroa S., Yao Z., Gao Y., Hines J., Samarasinghe K.T.G., Vogt L., Rosen N., Crews C.M. (2021). Mutant-selective degradation by BRAF-targeting PROTACs. Nat. Commun..

[54] Mattheisen J.M., Limberakis C., Ruggeri R.B., Dowling M.S., Am Ende C.W., Ceraudo E., Huber T., McClendon C.L., Sakmar T.P. (2023). Bioorthogonal Tethering Enhances Drug Fragment Affinity for G Protein-Coupled Receptors in Live Cells. J. Am. Chem. Soc..

[55] Mattheisen J.M., Wollowitz J.S., Huber T., Sakmar T.P. (2023). Genetic code expansion to enable site-specific bioorthogonal labeling of functional G protein-coupled receptors in live cells. Protein Sci..

[56] Corin K., Baaske P., Geissler S., Wienken C.J., Duhr S., Braun D., Zhang S. (2011). Structure and function analyses of the purified GPCR human vomeronasal type 1 receptor 1. Sci. Rep..

[57] Yoshida K., Nagatoishi S., Kuroda D., Suzuki N., Murata T., Tsumoto K. (2019). Phospholipid Membrane Fluidity Alters Ligand Binding Activity of a G Protein-Coupled Receptor by Shifting the Conformational Equilibrium. Biochemistry.

[58] Seidel S.A.I., Dijkman P.M., Lea W.A., van den Bogaart G., Jerabek-Willemsen M., Lazic A., Joseph J.S., Srinivasan P., Baaske P., Simeonov A. (2013). Microscale thermophoresis quantifies biomolecular interactions under previously challenging conditions. Methods.

[59] Poongavanam V., Atilaw Y., Siegel S., Giese A., Lehmann L., Meibom D., Erdelyi M., Kihlberg J. (2022). Linker-Dependent Folding Rationalizes PROTAC Cell Permeability. J. Med. Chem..

[60] Park S.H., Cheong C., Idoyaga J., Kim J.Y., Choi J.H., Do Y., Lee H., Jo J.H., Oh Y.S., Im W. (2008). Generation and application of new rat monoclonal antibodies against synthetic FLAG and OLLAS tags for improved immunodetection. J. Immunol. Methods.

[61] Enomoto M., Bunge M.B., Tsoulfas P. (2013). A multifunctional neurotrophin with reduced affinity to p75NTR enhances transplanted Schwann cell survival and axon growth after spinal cord injury. Exp. Neurol..

