# Supplementary material for: Targeted degradation of a constitutively active GPCR oncoprotein
Source: iScience. 2026 Nov 5;29(11):118116. doi: 10.1016/j.isci.2026.118116 (PMC13769065; doi:10.1016/j.isci.2026.118116)
Supplement: Supplementary file 1 [file mmc1.pdf]

iScience, Volume *29*

Supplemental information

Targeted degradation
# of a constitutively active
GPCR oncoprotein

Victoria R. Saca, Yuanhuang Chen, Colin Burdette, Thomas Huber, and Thomas P. Sakmar

1. Supplementary Figures

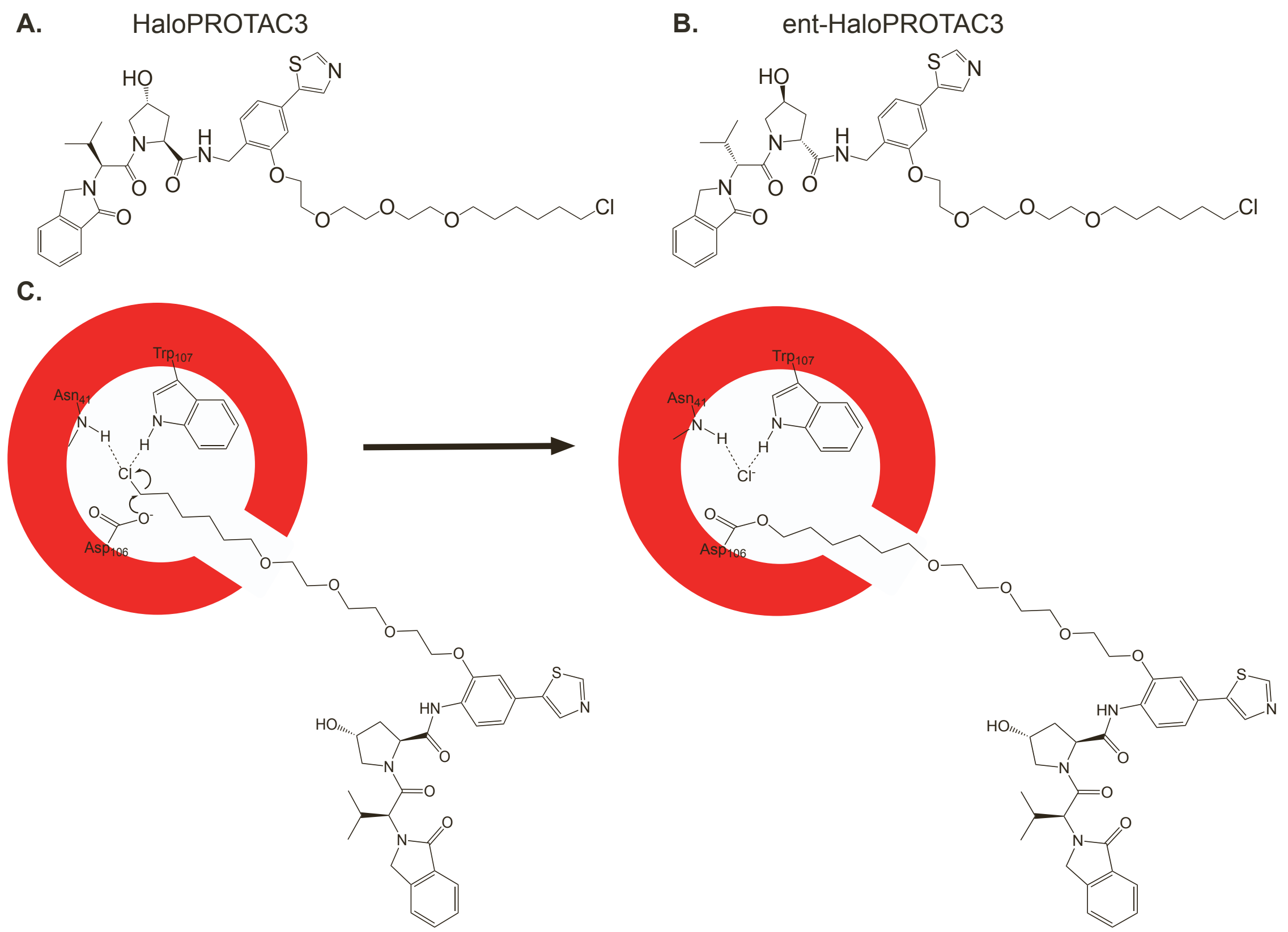

Figure S1. HaloPROTAC3 structure and self-labeling mechanism. A) Chemical structure of the HaloPROTAC3. **B)** Chemical structure of the enantiomer HaloPROTAC3. **C)** A nucleophilic displacement mechanism leads to the formation of a covalent ester bond between residue Asp106 of the HaloTag7 (red circle) and the chloroalkanyl moiety of the HaloPROTAC3.

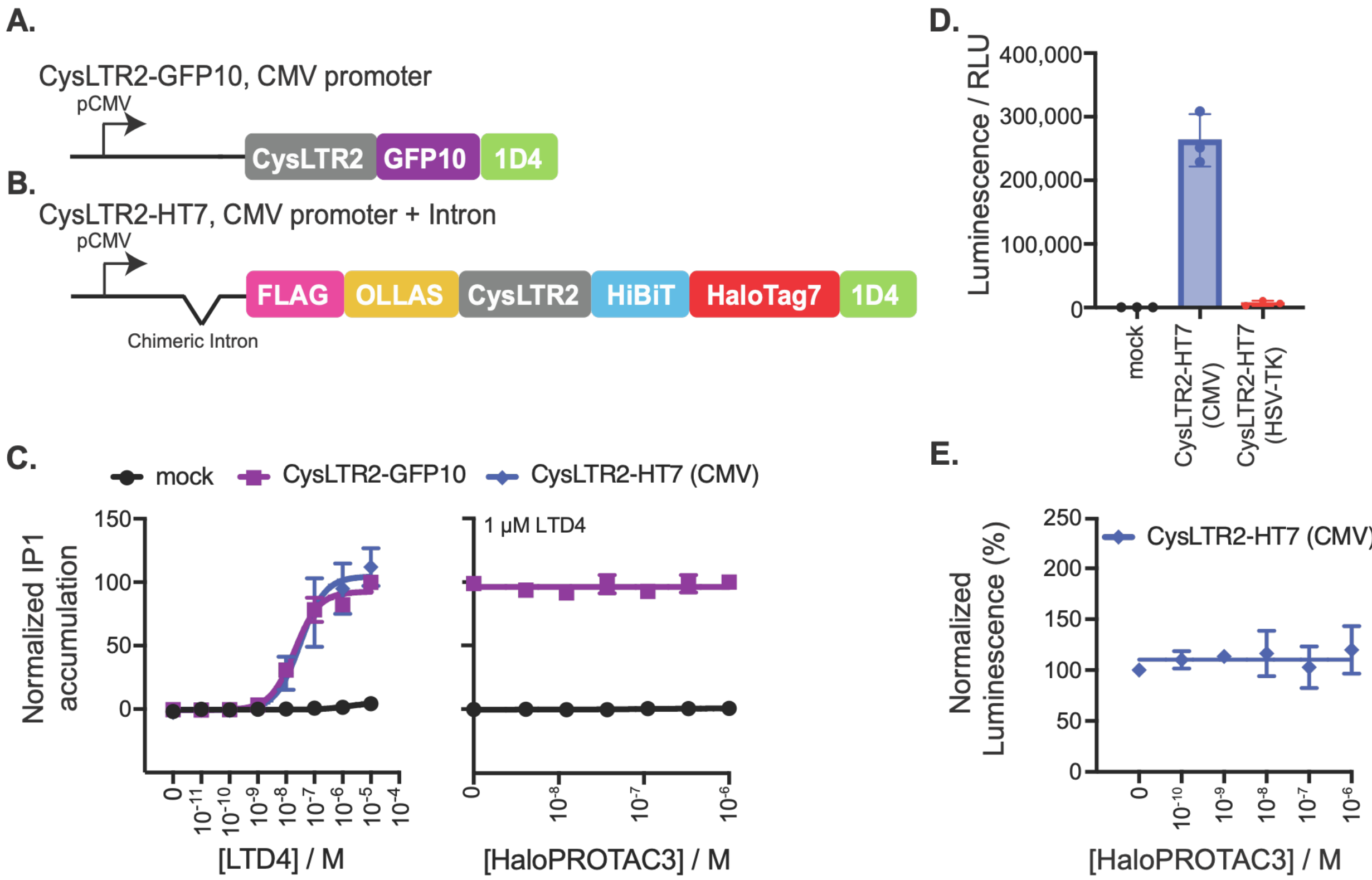

Figure S2: Characterization of strong promoter GPCR expression constructs. A) CysLTR2 with a C-terminal green fluorescent protein 10 (GFP10) and epitope tag (1D4) was used as a reference construct under the CMV promoter. **B**) A GPCR expression construct with two N-terminal epitope tags (FLAG and OLLAS), C-terminal bioluminescent nanoluciferase protein complementation tag (HiBiT), an engineered HaloTag7, and an epitope tag (1D4) under a strong promoter (CMV) was prepared for CysLTR2. **C)** Second messenger inositol monophosphate (IP_1) accumulation assay showed agonist dose-dependent IP_1 accumulation (*left*). Data were normalized to the highest concentration of agonist of the reference construct (CysLTR2-GFP10). IP_1 accumulation did not decrease with increasing concentrations of HaloPROTAC3 when CysLTR2-GFP10 was expressed under the strong CMV promoter. (*right*). Data were normalized to the condition without HaloPROTAC3. Data were plotted and fit to a sigmoidal or horizontal line model and compared using the Akaike Information Criterion. Each data point represents the mean signal from three independent experiments. **D)** HiBiT complementation luminescence assay was carried out using the strong CMV promoter expression construct CysLTR2-HT7. Each data point represents the mean signal from three technical replicates. **E)** HaloPROTAC3 did not affect the HiBiT complementation luminescence of the strong promoter expression construct. Data were normalized to receptor condition without treatment and plotted and fit to a three-parameter response or horizontal line model and compared using Akaike's Information Criterion. Each point represents the mean ± S.D. of normalized data from three individual experiments.

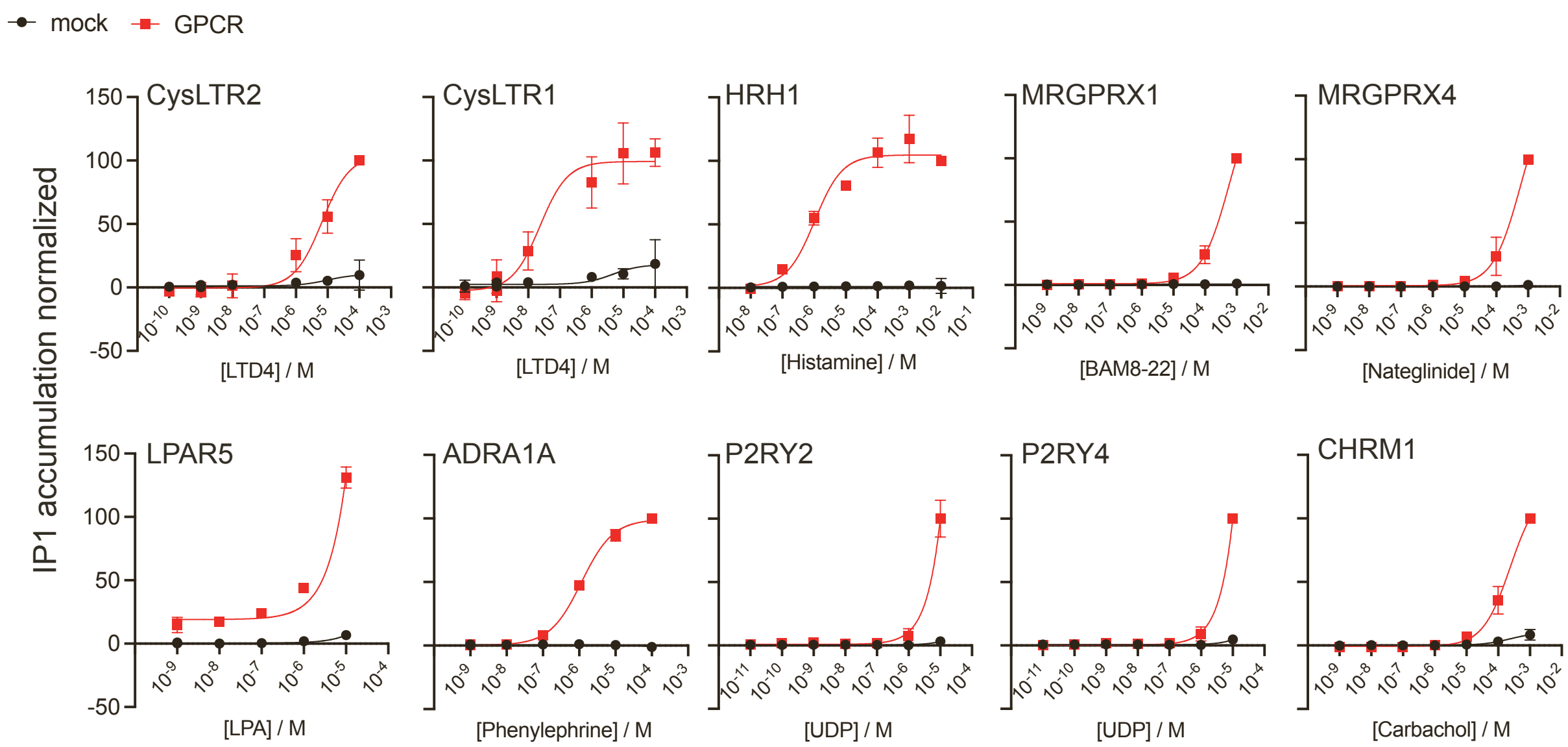

Figure S3: GPCR degron constructs signal in response to agonist treatment. GPCR expression constructs with two N-terminal epitope tags (FLAG and OLLAS), C-terminal bioluminescent nanoluciferase protein complementation tag (HiBiT), an engineered HaloTag7, and an epitope tag (1D4) under a weak promoter (HSV-TK) were prepared for ten GPCRs. IP_1 accumulation assays showed a dose-dependent increase in IP_1 treatment with agonist specific for each GPCR. Data were plotted and fit to a three-parameter response or horizontal line model and compared using Akaike's Information Criterion. Each data point represents the mean signal ± S.D. from three independent experiments.

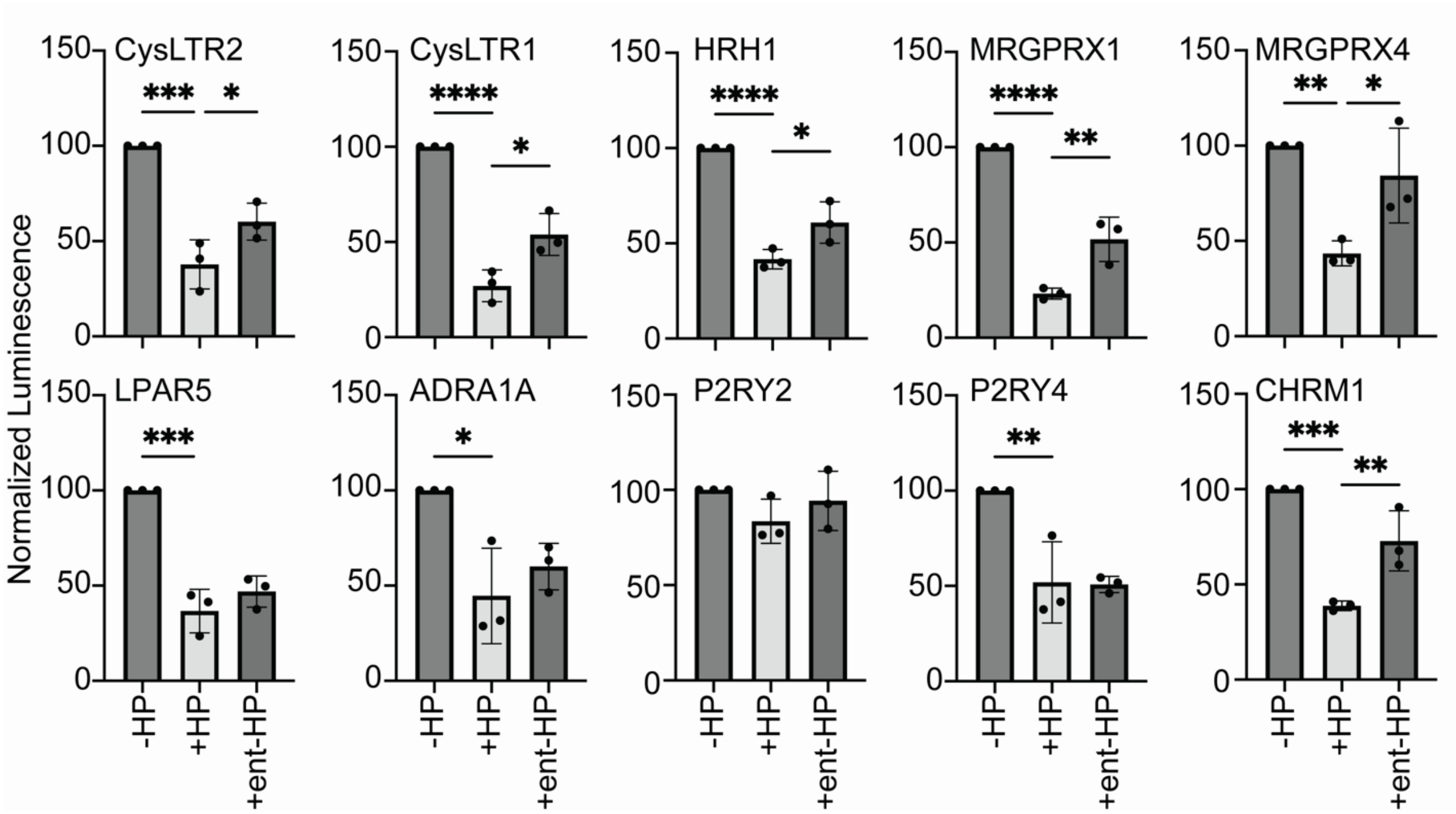

Figure S4: Luciferase complementation assay to measure levels of GPRC degron constructs after HaloPROTAC3 treatment. Normalized luminescence signals are shown for each of ten GPCR degron constructs after treatment with HaloPROTAC3 (HP) or enantiomer (ent-HP). Data were normalized and compared to the condition without treatment using a multiple comparisons one-way ANOVA. Each point represents the mean signal of three technical replicates. Data were normalized and compared to the condition without treatment using a multiple comparisons one-way ANOVA. Each data point represents the mean signal ± S.D. from three independent experiments. Conditions were compared using a multiple comparisons one-way ANOVA. Statistical significance is indicated as follows for all graphs: ns, $p > 0.05$; ∗, $p \leq 0.05$; ∗∗, $p \leq 0.01$; ∗∗∗, $p \leq 0.001$; and ∗∗∗∗, $p \leq 0.0001$.

1. **Raw Unprocessed Immunoblot.**

Table S1. Lane sample information for immunoblots that are source data for Figure 2B. Unprocessed immunoblots show the effects of HaloPROTAC3 and ent-HaloPROTAC3 on panel of GPCRs.

Lane number	Receptor	Condition
1	N/A (Ladder)	N/A
2	mock	vehicle
3	CysLTR2	vehicle
4	CysLTR2	HP
5	CysLTR2	ent-HP
6	CysLTR1	vehicle
7	CysLTR1	HP
8	CysLTR1	ent-HP
9	MRGPRX1	vehicle
10	MRGPRX1	HP
11	MRGPRX1	ent-HP
12	MRGPRX4	vehicle
13	MRGPRX4	HP
14	MRGPRX4	ent-HP
15	HRH1	vehicle
16	HRH1	HP
17	HRH1	ent-HP
18	N/A (Ladder)	vehicle
19	ADRA1A	vehicle
20	ADRA1A	HP
21	ADRA1A	ent-HP
22	CHRM1	vehicle
23	CHRM1	HP
24	CHRM1	ent-HP
25	LPAR5	vehicle
26	LPAR5	HP
27	LPAR5	ent-HP
28	P2RY2	vehicle
29	P2RY2	HP
30	P2RY2	ent-HP
31	P2RY4	vehicle
32	P2RY4	HP
33	P2RY4	ent-HP

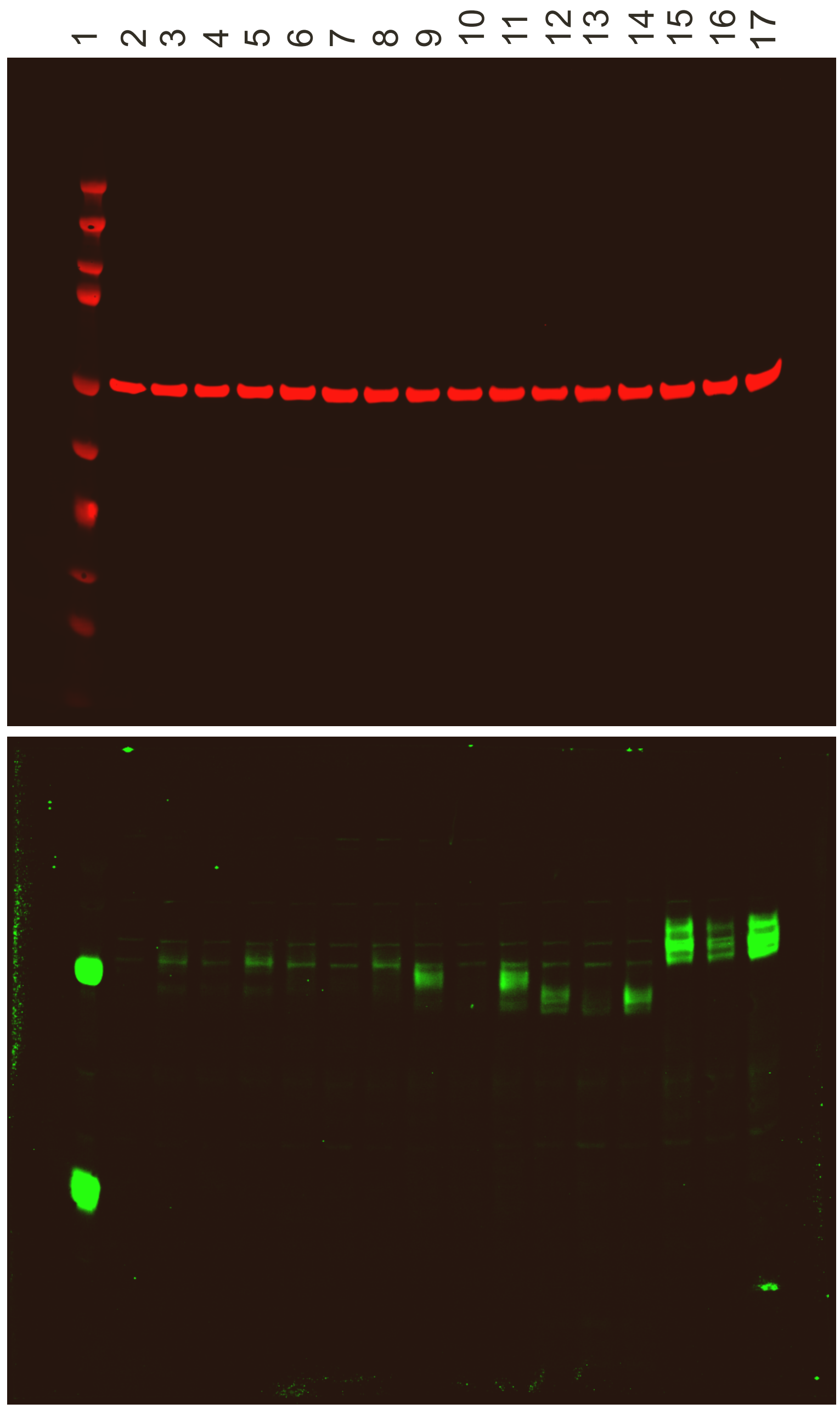

Figure S5. Source data Figure 2B (replicate 1, blot 1 of 6). Unprocessed immunoblot showing effects of HaloPROTAC3 and ent-HaloPROTAC3 on expression of panel of GPCRs. Red (top blot) is α-tubulin loading control and green (bottom blot) is total receptor as detected using OLLAS.

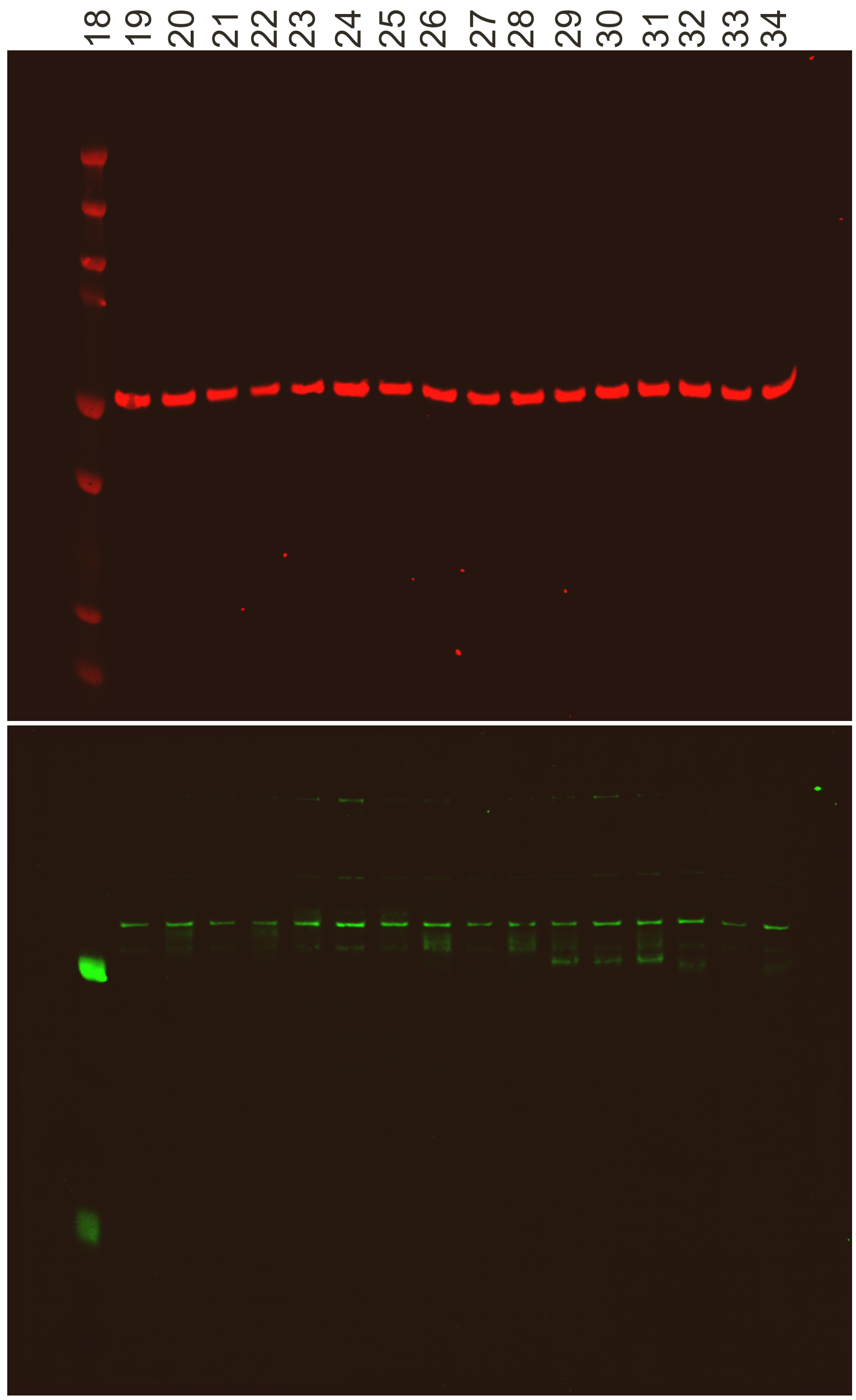

Figure S6. Source data Figure 2B (replicate 1, blot 2 of 6). Unprocessed immunoblot showing effects of HaloPROTAC3 and ent-HaloPROTAC3 on expression of panel of GPCRs. Red (top blot) is α-tubulin loading control and green (bottom blot) is total receptor as detected using OLLAS.

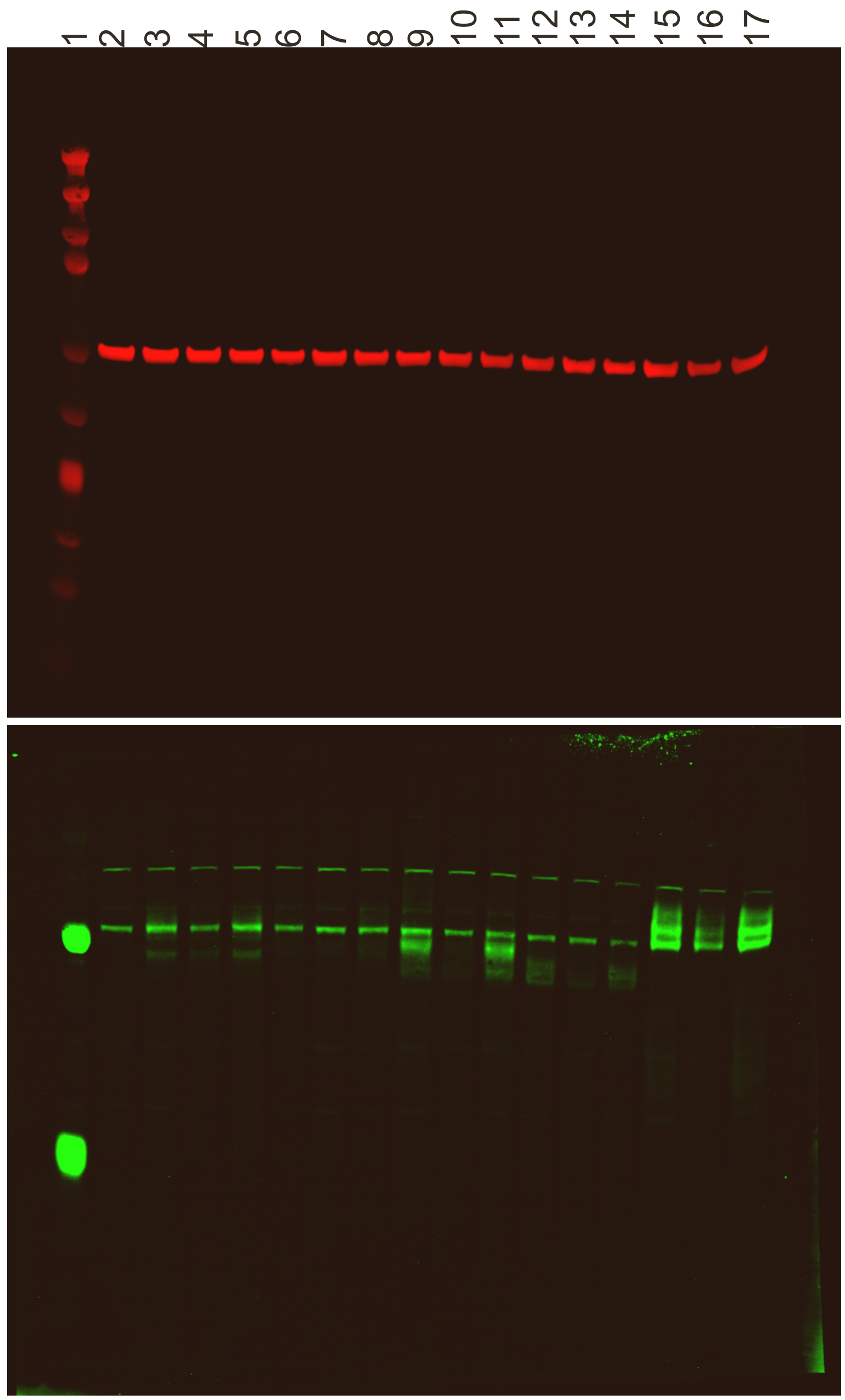

Figure S7. Source data Figure 2B (replicate 2, blot 3 of 6). Unprocessed immunoblot showing effects of HaloPROTAC3 and ent-HaloPROTAC3 on expression of panel of GPCRs. Red (top blot) is α-tubulin loading control and green (bottom blot) is total receptor as detected using OLLAS.

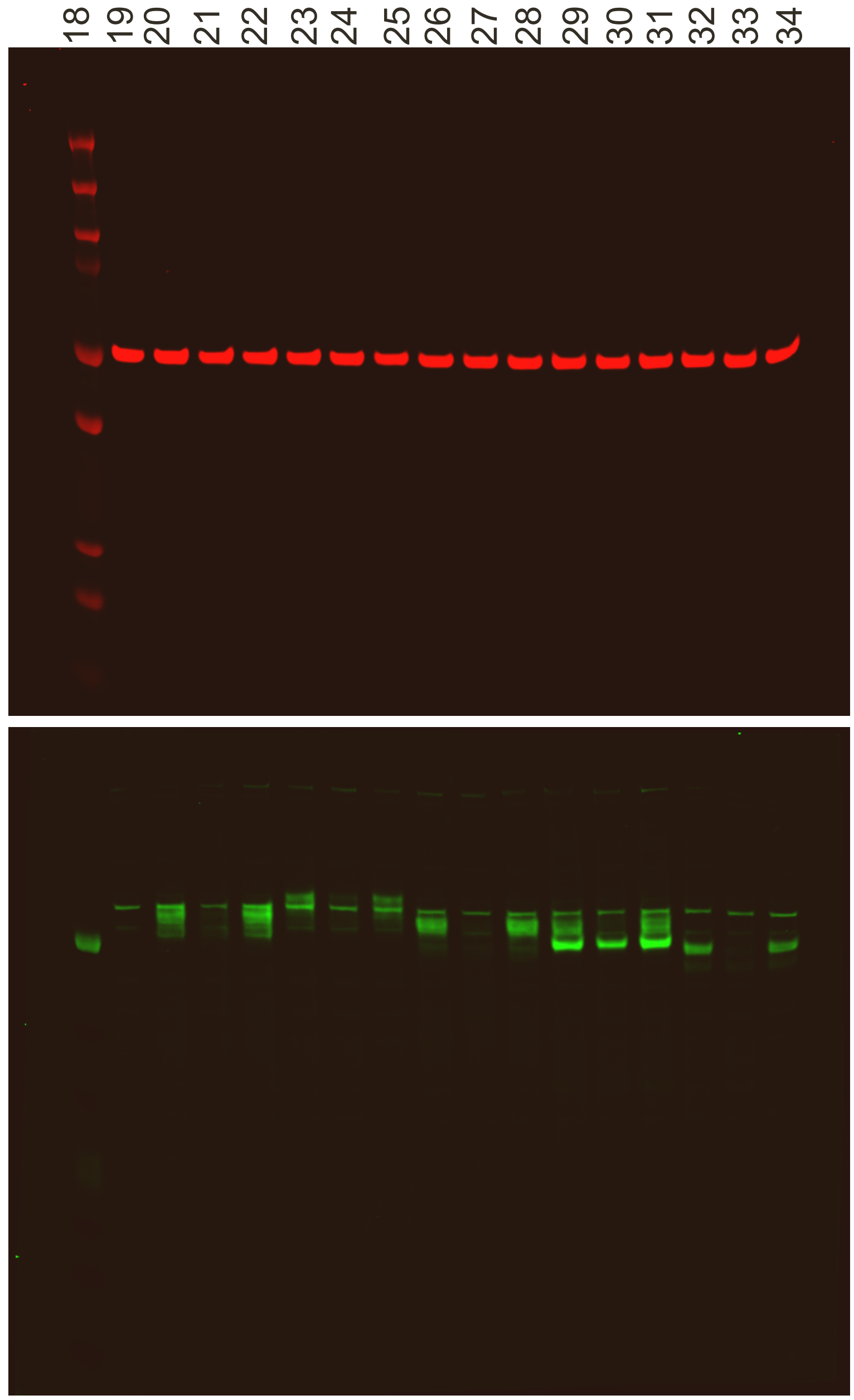

Figure S8. Source data Figure 2B (replicate 2, blot 4 of 6). Unprocessed immunoblot showing effects of HaloPROTAC3 and ent-HaloPROTAC3 on expression of panel of GPCRs. Red (top blot) is α-tubulin loading control and green (bottom blot) is total receptor as detected using OLLAS.

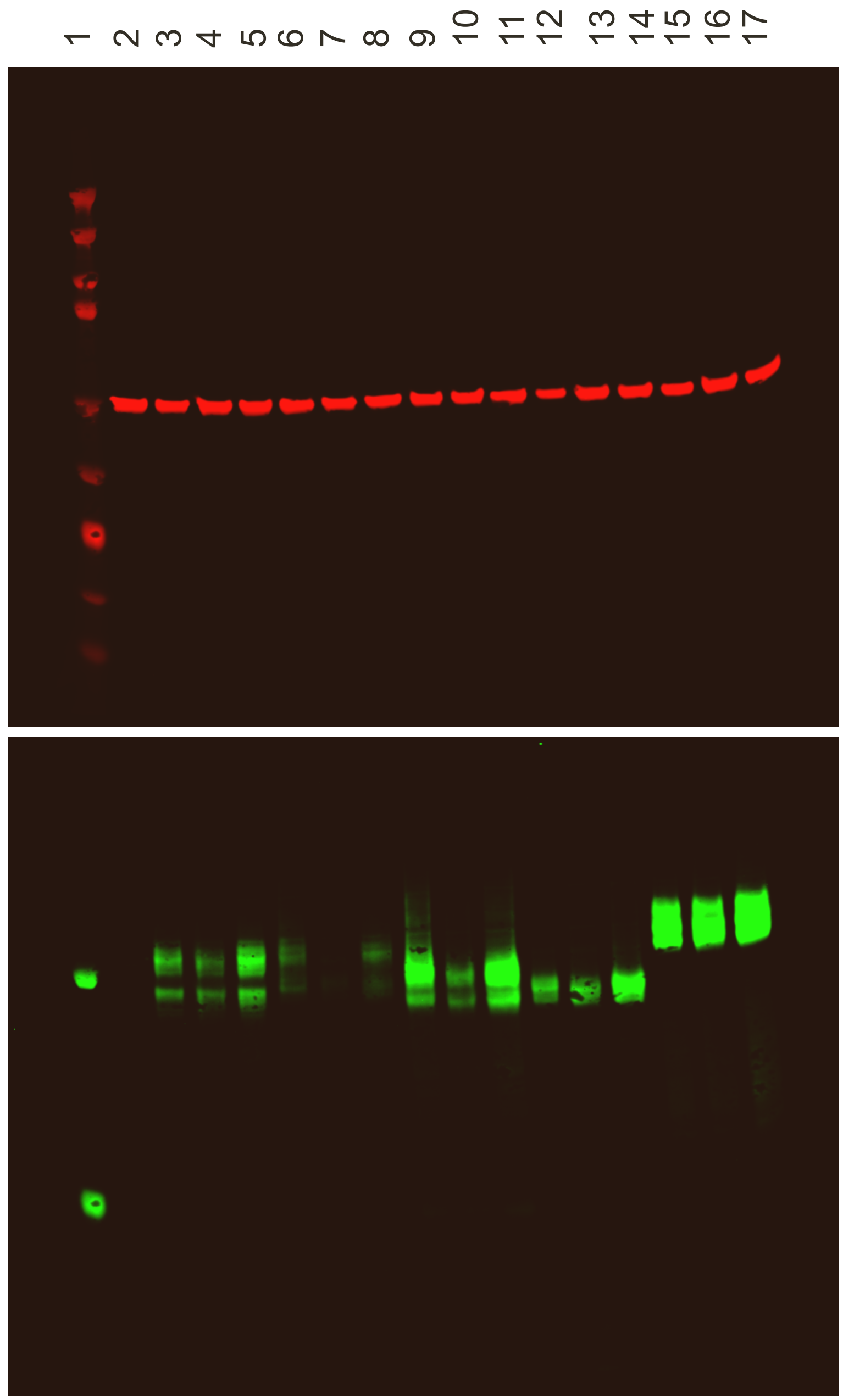

Figure S9. Source data Figure 2B (replicate 3, blot 5 of 6). Unprocessed immunoblot showing effects of HaloPROTAC3 and ent-HaloPROTAC3 on expression of panel of GPCRs. Red (top blot) is α-tubulin loading control and green (bottom blot) is total receptor as detected using OLLAS.

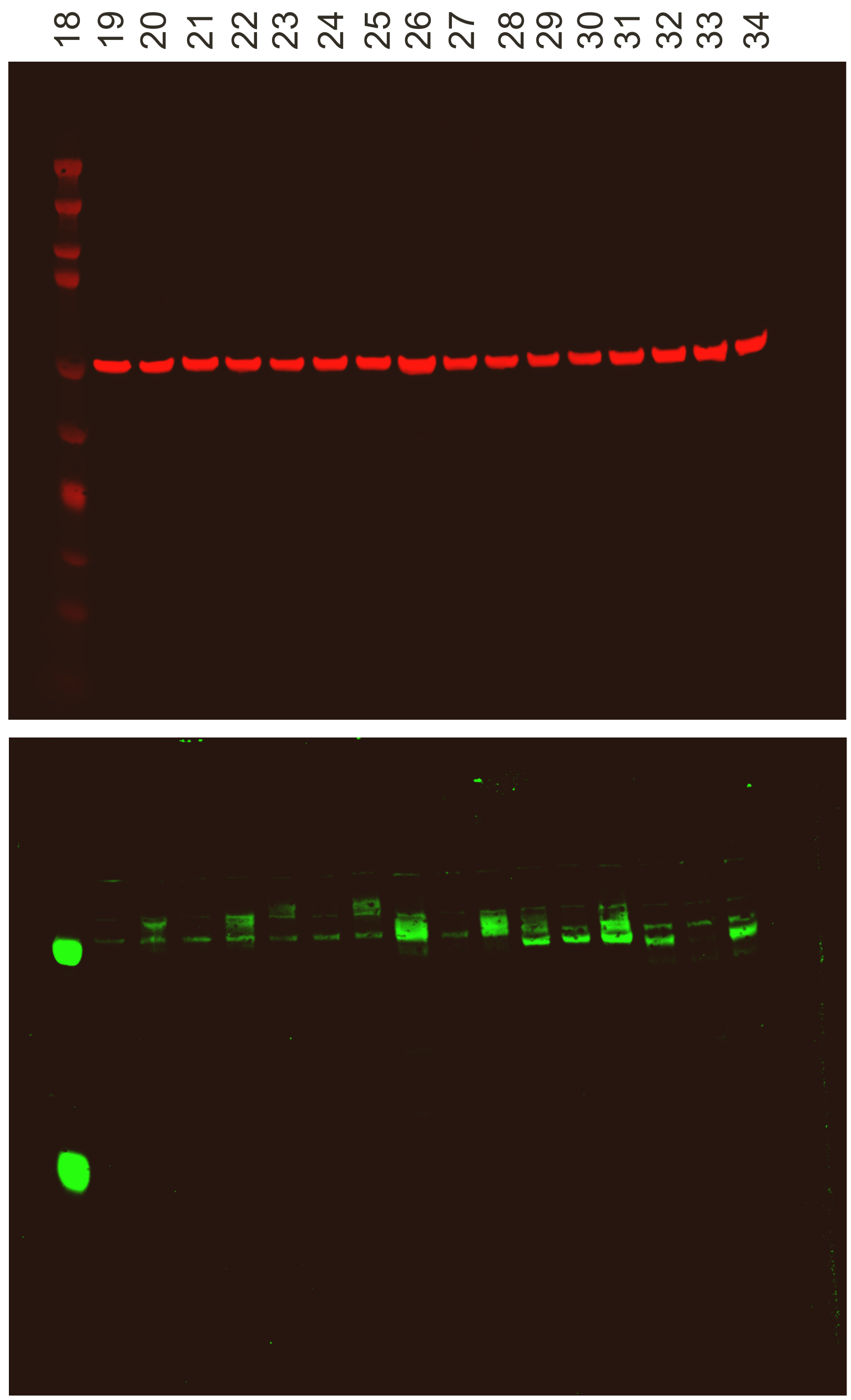

Figure S10. Source data Figure 2B (replicate 3, blot 6 of 6). Unprocessed immunoblot showing effects of HaloPROTAC3 and ent-HaloPROTAC3 on expression of panel of GPCRs. Red (top blot) is α-tubulin loading control and green (bottom blot) is total receptor as detected using OLLAS.

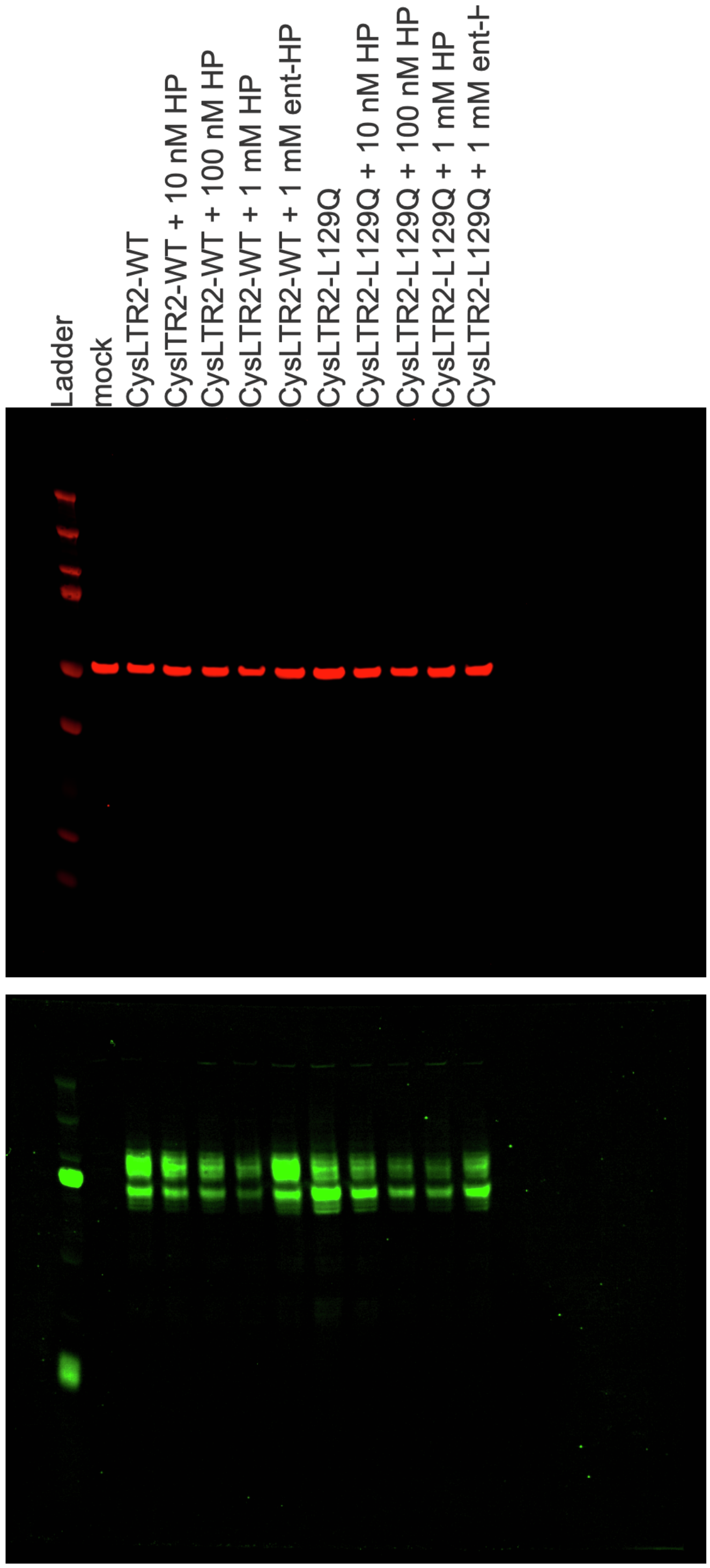

Figure S11. Source data Figure 3A (replicate 1). Unprocessed immunoblot showing effects of HaloPROTAC3 and ent-HaloPROTAC3 on expression of CysLTR2-WT and CysLTR2-L129Q. Red (top blot) is α-tubulin loading control and green (bottom blot) is total receptor as detected using OLLAS.

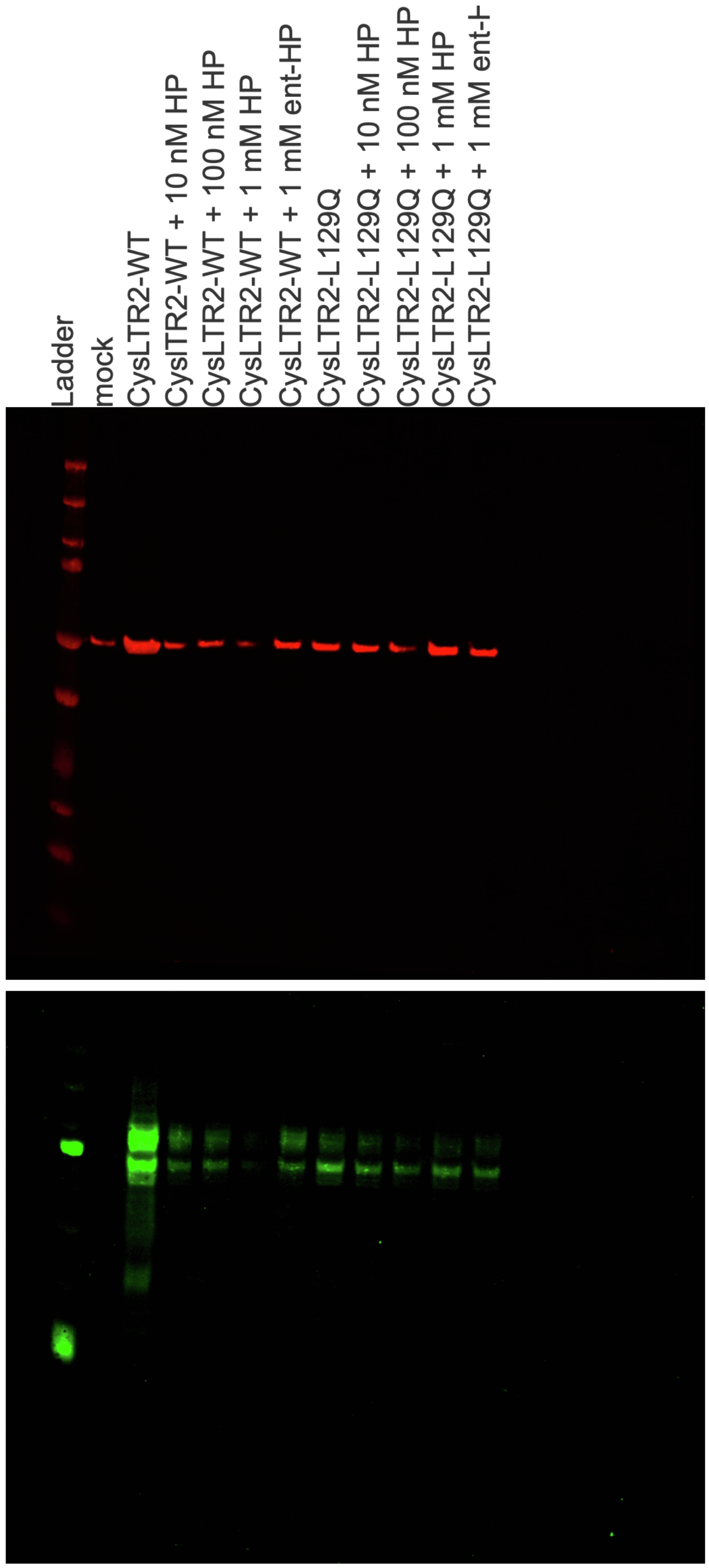

Figure S12. Source data Figure 3A (replicate 2). Unprocessed immunoblot showing effects of HaloPROTAC3 and ent-HaloPROTAC3 on expression of CysLTR2-WT and CysLTR2-L129Q. Red (top blot) is α-tubulin loading control and green (bottom blot) is total receptor as detected using OLLAS.

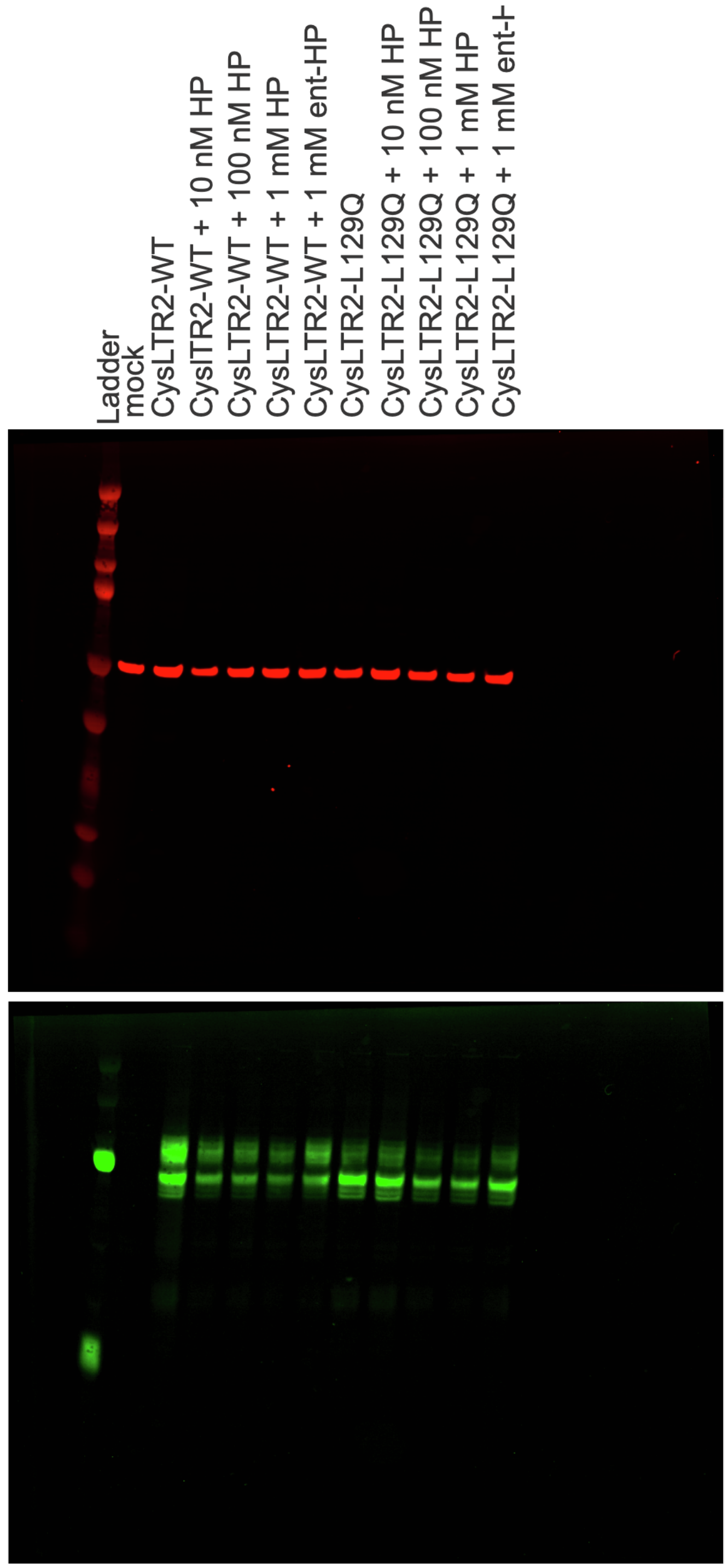

Figure S13. Source data Figure 3A (replicate 3). Unprocessed immunoblot showing effects of HaloPROTAC3 and ent-HaloPROTAC3 on expression of CysLTR2-WT and CysLTR2-L129Q. Red (top blot) is α-tubulin loading control, and green (bottom blot) is total receptor as detected using OLLAS.

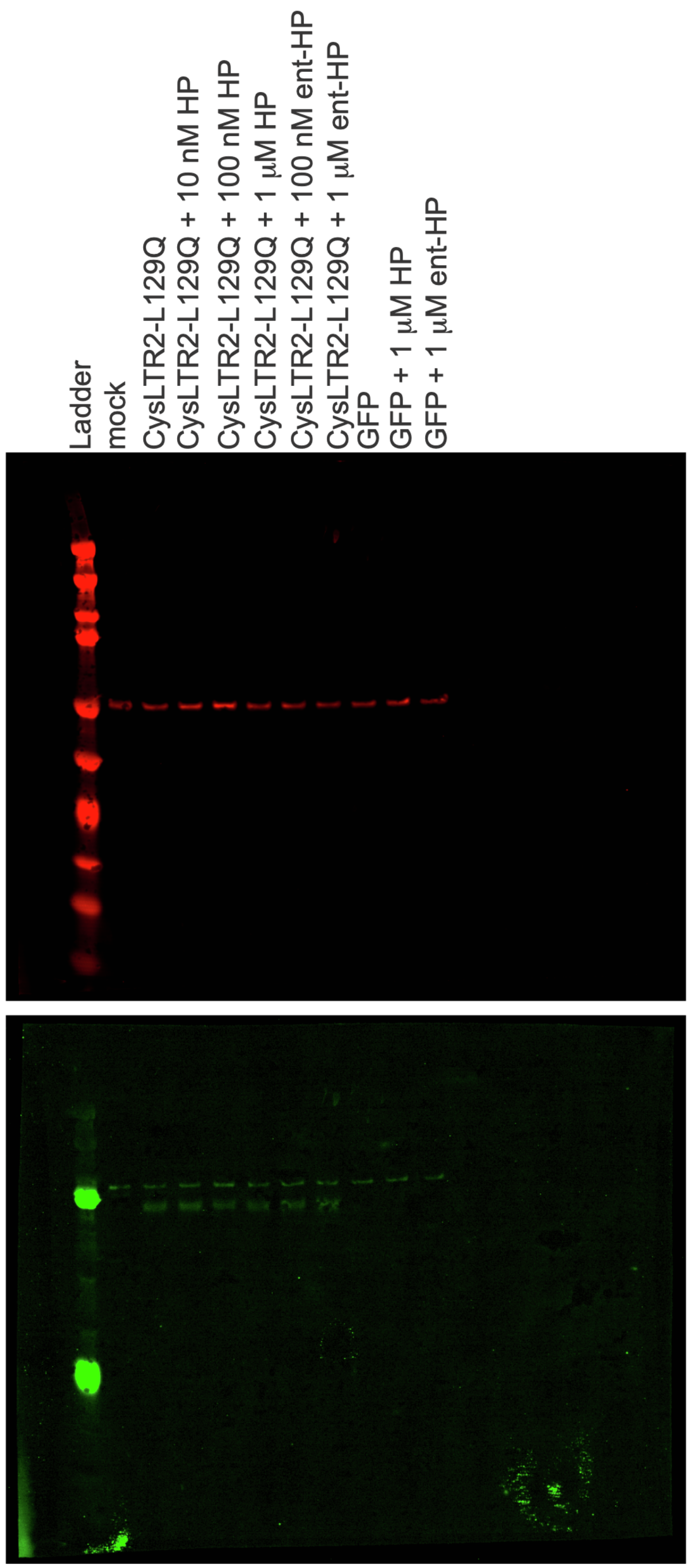

Figure S14. Source data Figure 6A (replicate 1). Unprocessed immunoblot showing effects of HaloPROTAC3 and ent-HaloPROTAC3 on expression of CysLTR2-L129Q in melanocytes. Red (top blot) is α-tubulin loading control, and green (bottom blot) is total receptor as detected using OLLAS.

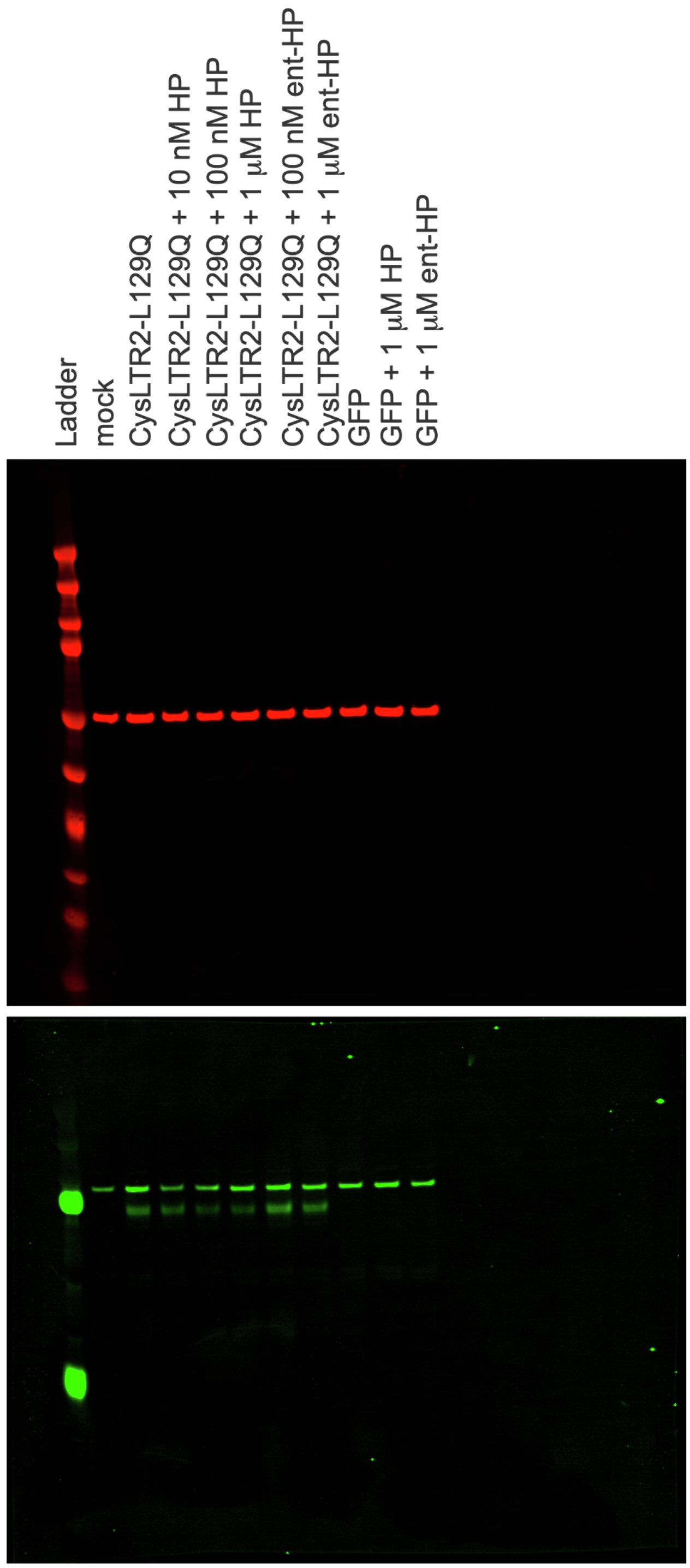

Figure S15. Source data Figure 6A (replicate 2). Unprocessed immunoblot showing effects of HaloPROTAC3 and ent-HaloPROTAC3 on expression of CysLTR2-L129Q in melanocytes. Red (top blot) is α-tubulin loading control, and green (bottom blot) is total receptor as detected using OLLAS.

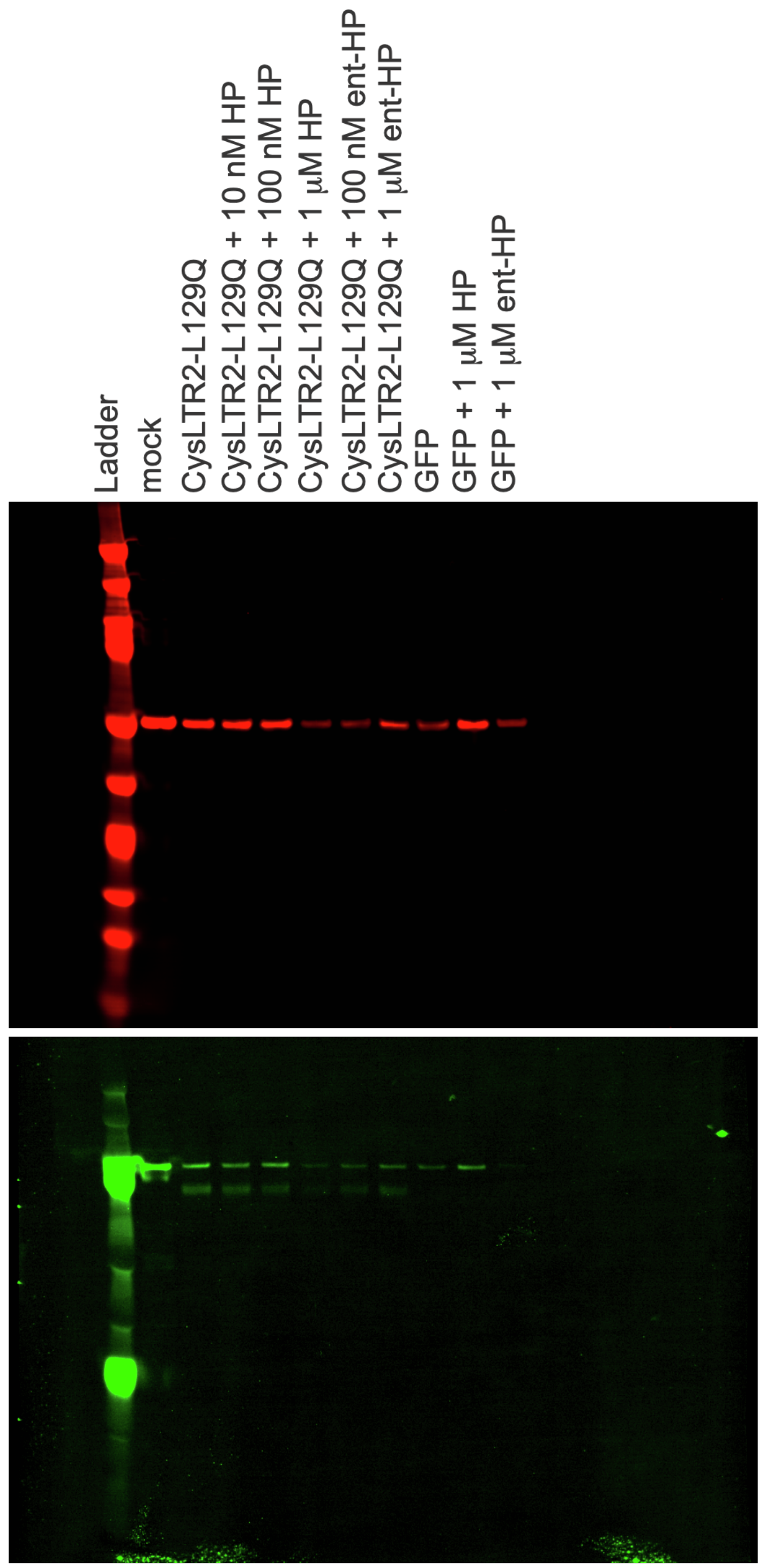

Figure S16. Source data Figure 6A (replicate 3). Unprocessed immunoblot showing effects of HaloPROTAC3 and ent-HaloPROTAC3 on expression of CysLTR2-L129Q in melanocytes. Red (top blot) is α-tubulin loading control, and green (bottom blot) is total receptor as detected using OLLAS.

Table S2: NEBuilder HiFi DNA assembly primers and oligos used for GPCR panel

Purpose of Primer	Sequence of Primers (5' to 3')
Primer pair that anneals to TH2001 (HSV-TK-SP-FLAG-OLLAS-CysLTR2-HiBiT-HT7-1D4) excluding CysLTR2 to create the backbone for the panel of GPCRs	cggtgaattcGGTACCGTGAGCGGCTGG
	cgatgctggcGGTGTCATCTCCTGCAGGACC
Primer pair that anneals to DuET library construct including only the receptor to create the insert for the panel of GPCRs	gagatgacaccGCCAGCATCGATATG
	tcacggtaccGAATTCACCGGTGCGTCC

Table S3: QuickChange Lightning Site Directed Mutagenesis primers to produce CysLTR2-L129Q mutant

Purpose of Primer	Sequence of Primers (5' to 3')
Forward Primer	cagctccatctacttccaaaccgtgctgagcgtgg
Reverse Primer	ccacgctcagcacggtttggaagtagatggagctg

Table S4: NEBuilder HiFi DNA assembly primers and oligos used for lentiviral production construct

Purpose of Primer	Sequence of Primers (5' to 3')
Primer pair that anneals to pLV-eGFP excluding the GFP to create the backbone for the lentiviral CysLTR2-L129Q	ccctgcctaaaagcggccgcgtcgacaa
	aatcgctagcagctctgcttatatagacctcccacc
Primer pair that anneals to TH2001 (HSV-TK-SP-FLAG-OLLAS-CysLTR2-HiBiT-HT7-1D4) from SP to 1D4 to create the insert for the panel of GPCRs	aagcagagctgctagcgattcttctgacacaacag
	gcggccgcttttaggcaggggccacctg

Table S5: Fitting parameters for experimental agonist dose response curves. Parameters for the sigmoidal *versus* horizontal line model are shown below. Data showing no significant dose-response are represented by a horizontal line and are defined by the mean only. The Akaike Information Criterion was used to determine the best-fit model for each data set.

	Best Fit Values					95% Confidence Intervals		
	Bottom	Top	logIC50	Amplitude	Mean	Bottom	Top	logIC50
Figure 1C (normalized)								
mock	0.5071	3.47	-7.268	2.962		-0.600 to 1.576		-9.030 to 3.919
CysLTR2-GFP10	-0.7881	92.61	-7.703	93.40		-4.378 to 2.779	87.95 to 97.37	-7.849 to -7.553
CysLTR2-HT7	0.0375	27.95	-6.125	27.91		-4.431 to 4.243	16.42 to 55.75	-7.289 to -4.997
Figure S2C left (normalized)								
mock	-0.3891	4.983	-5.811	5.372		-1.258 to 0.453		-7.540 to 6.255
CysLTR2-GFP10	-0.7881	92.61	-7.703	93.40		-4.378 to 2.779	87.95 to 97.37	-7.849 to -7.553
CysLTR2-HT7 (CMV)	-0.7126	104.6	-7.495	105.3		-8.780 to 7.194	93.32 to 116.6	-7.827 to -7.163

Table S6: Fitting parameters for experimental data from HaloPROTAC3 dose response curves for weak and strong promoters. Parameters for a three-parameter inhibitor response *versus* the horizontal line model are shown below. Data showing no significant dose-response are represented by a horizontal line and are defined by the mean only. The Akaike Information Criterion was used to determine the best-fit model for each data set.

	Best Fit Values					95% Confidence Intervals		
	Bottom	Top	logIC50	Amplitude	Mean	Bottom	Top	logIC50
Figure 1E (normalized)								
CysLTR2-HT7	139.5	34.38	2.73	105.1		109.3 to 170.9	-286.4 to 87.32	1.690 to 4.570
Figure S2C (normalized)								
CysLTR2-GFP10					96.28			
Figure S2E (normalized)								
CysLTR2-HT7 (CMV)					110.4			

Table S7: Fitting parameters for experimental agonist dose response curves for GPCRs in degron library. Parameters for the sigmoidal *versus* horizontal line model are shown below. Data showing no significant dose-response are represented by a horizontal line and are defined by the mean only. The Akaike Information Criterion was used to determine the best-fit model for each data set.

	Best Fit Values					95% Confidence Intervals		
Figure S2 (normalized)	Bottom	Top	logEC50	Amplitude	Mean	Bottom	Top	logEC50
mock	1.163	10.38	-5.026	9.128		0.3531 to 3.294		-8.074 to 6.807
CysLTR2	-0.678	104	-5.18	104.7		-5.605 to 4.149	89.36 to 120.0	-5.520 to -4.903
mock	1.972	16.73	-5.662	14.76		-2.198 to 5.872		-7.768 to 5.463
CysLTR1	-1.776	106.8	-6.588	108.6		-9.774 to 6.094	96.05 to 118.1	-6.886 to -6.286
mock					1.09			
HRH1	0.8375	104.7	-5.983	103.8		-7.670 to 9.194	97.63 to 111.8	-6.250 to -5.700
mock	0.1652	0.823	-5.855	0.6577		0.0614 to 0.5859	0.3841 to 5578	
MRGPRX1	1.017	156.5	-3.243	155.5		-0.1738 to 2.207	142.4 to 177.7	-3.353 to -3.111
mock	0.029	1.2631	9.785	1.2341		-0.1800 to 0.2501		
MRGPRX4	0.6642	157	-3.241	156.4		-1.706 to 3.032	132.3 to 216.2	-3.444 to -2.938
mock	0.0903	10.96	-4.564	10.87		-1.026 to 1.198		-5.264 to 7.437
LPAR5	11.72	139.6	-4.347	127.9		9.516 to 13.92	123.5 to 169.2	-4.520 to -4.117
mock	0.2648	-0.771	11.24					
ADRA1A	0.8704	102.3	-4.35	101.4		-3.487 to 5.168	91.23 to 114.5	-4.610 to -4.110
mock	1.121	12.07	-3.586	10.94		-0.8985 to 3.100		-6.024 to 8.362
P2RY2	0.2575	100	8.939			-1.358 to 1.635		
mock	1.054	11.93	-3.582	10.87		-0.5829 to 2.667		-5.175 to 8.679
P2RY4	-0.406	812.7	-2.149	813.1		-2.126 to 1.302		
mock	0.0922	10.37	-3.621	10.28		-0.6457 to 0.8258	7.437 to 28.24	-4.122 to -2.626
CHRM1	-0.597	124.1	-3.619	124.7		-2.434 to 1.239	115.4 to 134.5	-3.726 to -3.501

Table S8. Calculations for immunoblot analysis of screen of GPCR degron library. The 800-nm signal is the total receptor expression and the 700-nm signal is the α-tubulin loading control. Lane normalization factor (LNF) was calculated for each lane by dividing the 700-nm loading control signal intensity of the lane by the highest loading control signal intensity. Signals in the 800-nm channel were normalized (Norm signal) by dividing the signal intensity by the calculated LNF. The percent signal decrease was divided by the condition without drug and converted to a percentage.

	Figure 2B		Replicate 1				Replicate 2				Replicate 3			
	Receptor	Condition	700-nm Signal	800-nm signal	Norm signal	Percent Signal Decrease	700-nm Signal	800-nm signal	Norm signal	Percent Signal Decrease	700-nm Signal	800-nm signal	Norm signal	Percent Signal Decrease
Blot 1	mock	no drug	6,920	-0.122	-0.20		6,750	-2.61	-2.72		8,640	-1.78	-1.78	
	CysLTR2	no drug	8,020	4.79	6.72	100.00	7,040	13.6	13.59	100.00	6,910	450	562.95	100.00
	CysLTR2	HP	7,600	3.05	4.52	67.26	6,570	9.02	9.66	71.08	7,770	193	215.06	38.20
	CysLTR2	ent-HP	8,750	4.82	6.19	92.11	6,050	15.4	17.88	131.52	7,490	603	696.02	123.64
	CysLTR1	no drug	9,370	7.03	8.44	100.00	5,800	7.34	8.91	100.00	7,660	265	318.94	100.00
	CysLTR1	HP	8,880	3.24	4.11	48.70	6,100	4.81	5.55	62.30	6,950	46.7	58.06	18.21
	CysLTR1	ent-HP	8,460	7.98	10.60	125.59	6,130	9.69	11.12	124.88	7,450	314	363.91	114.10
	MRGPRX1	no drug	7,440	87.2	132.00	100.00	5,700	85.1	105.08	100.00	6,830	1,570	1989.38	100.00
	MRGPRX1	HP	7,240	0.562	0.87	0.66	5,560	6.21	7.87	7.49	7,030	483	593.95	29.86
	MRGPRX1	ent-HP	6,960	116	187.00	141.67	4,950	96.3	137.15	130.52	7,430	1,850	2152.09	108.18
	MRGPRX4	no drug	7,130	63.4	100.00	100.00	5,190	43.8	59.38	100.00	4,690	898	1656.82	100.00
	MRGPRX4	HP	7,630	24.1	35.50	35.50	5,730	13.9	17.12	28.84	7,160	608	733.78	44.29
	MRGPRX4	ent-HP	7,690	114	167.00	167.00	4,610	36.6	55.81	93.99	7,530	1,940	2226.36	134.37
	HRH1	no drug	9,290	108	131.00	100.00	6,270	116	130.59	100.00	6,640	1,370	1854.43	100.00
	HRH1	HP	9,660	16.9	19.60	14.96	4,250	193	31.98	24.49	8,450	870	889.84	47.98
	HRH1	ent-HP	11,200	204	204.00	155.73	4,460	174	274.19	209.96	7,820	1,600	2383.19	128.51
Blot 2	mock	no drug	7,760	0.235	0.28		9,170	26.3	30.48		9,260	5.89	7.11	
	ADRA1A	no drug	7,470	22	27.62	100.00	10,600	352	352.16	100.00	8,320	35.3	47.40	100.00
	ADRA1A	HP	5,570	2.13	3.59	13.00	8,570	38.2	47.37	13.45	8,330	5.71	7.66	16.16
	ADRA1A	ent-HP	4,250	19.9	44.04	159.43	9,280	507	579.77	164.63	8,490	43.5	57.20	120.68
	CHRM1	no drug	5,910	14.5	23.06	100.00	8,680	193	235.72	100.00	7,670	38	55.40	100.00
	CHRM1	HP	9,390	3.37	3.37	14.62	8,510	65.9	82.33	34.93	8,370	10.3	13.80	24.91
	CHRM1	ent-HP	7,100	12	15.87	68.80	7,210	167	245.67	104.22	8,680	51.3	66.10	119.31
	LPAR5	no drug	7,830	59.8	71.69	100.00	8,170	513	667.77	100.00	11,200	125	125.00	100.00
	LPAR5	HP	6,510	7.68	11.06	15.43	8,020	57.3	75.74	11.34	7,400	2.77	4.19	3.35
	LPAR5	ent-HP	7,080	51.2	67.89	94.69	9,070	560	656.47	98.31	6,370	108	190.00	152.00
	P2RY2	no drug	6,650	16.4	23.14	100.00	9,320	364	414.43	100.00	6,560	32.8	55.90	100.00

	P2RY2	HP	8,360	10.5	11.83	51.10	9,350	97.5	110.75	26.72	6,340	19.4	34.10	61.00
	P2RY2	ent-HP	8,230	17.8	20.28	87.61	10,100	365	385.71	93.07	7,950	34.4	48.30	86.40
	P2RY4	no drug	8,880	22.9	24.19	100.00	9,600	261	288.59	100.00	8,560	66.1	86.40	100.00
	P2RY4	HP	6,160	1.02	1.55	6.43	9,840	14.3	15.49	5.37	9,440	6.59	7.80	9.03
	P2RY4	ent-HP	7,040	11	14.72	60.85	9,240	281	322.86	111.88	9,030	84.3	104.00	120.37

Table S9. Fitting parameters for experimental HaloPROTAC3 (HP) dose response curves for GPCRs in degron library. Parameters for a three-parameter inhibitor response *versus* a horizontal line model are shown below. Data showing no significant dose-response are represented by a horizontal line and are defined by the mean only. The Akaike Information Criterion were used to determine the best fit model for each data set.

Figure 2C (normalized)

	Best Fit Values					95% Confidence Intervals		
	Bottom	Top	logIC50	Amplitude	Mean	Bottom	Top	logIC50
mock + HP					-0.711			
CysLTR2 + HP	7.243	105.8	-1.125	98.6		-3.234 to 16.43	97.57 to 115.2	-1.335 to -0.9167
mock + ent-HP					0.3482			
CysLTR2 + ent-HP					114.1			
mock + HP					-1.626			
CysLTR1 + HP	13.97	114.7	-1.434	100.7		-0.8760 to 26.86	97.53 to 139.4	-1.825 to -1.072
mock + ent-HP					-2.713			
CysLTR1 + ent-HP					81.38			
mock + HP					0.7994			
HRH1 + HP	29.28	124.7	-1.747	95.39		14.72 to 41.83	97.55 to 200.9	-2.426 to -1.230
mock + ent-HP					0.9169			
HRH1 + ent-HP					81.8			
mock + HP	-1.609	-0.123	13					
MRGPRX1 + HP	12.46	103.4	-1.161	90.9		-11.26 to 30.01	86.55 to 125.6	-1.641 to -0.6823
mock + ent-HP					0.1473			
MRGPRX1 + ent-HP					103.9			
mock + HP					0.1626			
MRGPRX4 + HP	13.8	103.4	-0.9173	89.57		-4.863 to 27.59	94.32 to 114.0	-1.244 to -0.5893
mock + ent-HP					-0.214			
MRGPRX4 + ent-HP					98.32			
mock + HP					0.1379			
LPAR5 + HP	50.37	105.6	-1.297	55.27		38.44 to 59.78	94.90 to 121.2	-1.785 to -0.8365
mock + ent-HP					0.5834			
LPAR5 + ent-HP					99.3			
mock + HP	-0.498	0.1061	12.78					
ADRA1A + HP	8.328	105.3	-1.05	96.96		-11.74 to 23.64	92.73 to 120.2	-1.400 to -0.6901
mock + ent-HP					0.2067			
ADRA1A + ent-HP					101.1			
mock + HP					-2.118			
P2RY2 + HP	24.5	120.2	-1.707	95.73		14.35 to 33.82	100.9 to 151.7	-2.088 to -1.372
mock + ent-HP					4.62			
P2RY2 + ent-HP					107.8			
mock + HP					2.782			
P2RY4 + HP	32.33	111	-1.37	78.69		24.46 to 39.37	101.9 to 122.1	-1.618 to -1.128
mock + ent-HP					2.165			
P2RY4 + ent-HP					99.33			
mock + HP	-0.405	0.0775	13.91					
CHRM1 + HP	26.95	108.7	-0.6444	81.72		-11.13 to 46.66	99.63 to 118.8	-1.055 to -0.1633
mock + ent-HP					0.0996			
CHRM1 + ent-HP					103.5			

Table S10. Calculations for immunoblot analysis of CysLTR2-WT and CysLTR2-L129Q after HaloPROTAC3 (HP) treatment. The 800-nm signal is the total receptor expression and the 700-nm signal is the α-tubulin loading control. Lane normalization factor (LNF) was calculated for each lane by dividing the 700-nm loading control signal intensity of the lane by the highest loading control signal intensity. Signals in the 800-nm channel were normalized (Norm signal) by dividing the signal intensity by the calculated LNF. The percent signal decrease was divided by the condition without drug and converted to a percentage.

			CysLTR2-WT					CysLTR2-L129Q				
	Figure 3A	mock	no HP	10 nM HP	100 nM HP	1 µM HP	1 µM ent-HP	no HP	10 nM HP	100 nM HP	1 µM HP	1 µM ent-HP
Replicate 1	700-nm signal	5,870	5,290	5,330	5,010	4,120	6,280	8,910	6,400	4,890	5,920	6,100
	800-nm signal	0.941	284	229	89.9	61.7	349	108	42.9	51.5	40.7	87.6
	Norm signal	1.43	478	383	160	133	495	108	59.8	94	61.2	126
	Percent Signal Decrease		100	80.13	33.47	27.82	103.56	100.00	55.37	87.04	56.67	116.67
Replicate 2	700-nm signal	1,430	7,020	1,700	2,290	866	3,400	3,510	2,370	1,970	5,660	4,390
	800-nm signal	0.451	231	36.7	28.8	5.02	58.1	21.4	8.85	2.52	12.3	17.4
	Norm signal	2.21	231	152	88.2	40.8	120	42.9	26.2	8.99	15.3	27.8
	Percent Signal Decrease		100	65.80	38.18	17.66	51.95	100.00	61.07	20.96	35.66	64.80
Replicate 3	700-nm signal	9,310	12,600	6,190	8,160	7,770	7,920	7,310	9,030	7,970	7,870	9,040
	800-nm signal	0.236	468	199	83.5	109	329	64.9	69.5	46.2	26.7	71.9
	Norm signal	0.318	468	404	129	177	522	112	96.7	72.9	42.6	99.9
	Percent Signal Decrease		100	86.32	27.56	37.82	111.54	100.00	86.34	65.09	38.04	89.20

Table S11: Fitting parameters for experimental HaloPROTAC3 (HP) dose response curves for CysLTR2-WT and CysLTR2-L129Q. Parameters for a three-parameter inhibitor response *versus* a horizontal line model are shown below. Data showing no significant dose-response are represented by a horizontal line and are defined by the mean only. The Akaike Information Criterion was used to determine the best-fit model for each data set.

Figure 3B Left + HP (normalized)

	Best Fit Values					95% Confidence Intervals		
	Bottom	Top	logIC50	Amplitude	Mean	Bottom	Top	logIC50
Figure 3B Left + HP (normalized)								
mock	14.96	-1.13	0.5674	-16.09				
CysLTR2 + LTD4	-8.69	101.3	0.1359	110			94.42 to 108.6	
CysLTR2 - LTD4					-1.57			
CysLTR2-L129Q + LTD4	20.11	77.45	0.0735	57.34			70.16 to 87.23	
CysLTR2-L129Q - LTD4	17.07	40.97	-0.43	23.9			36.68 to 46.35	
Figure 3B Right + ent-HP (normalized)								
mock					5.602			
CysLTR2 + LTD4					95.77			
CysLTR2 - LTD4					6.85			
CysLTR2-L129Q + LTD4					77.39			
CysLTR2-L129Q - LTD4					49.45			

Table S12. Calculations for immunoblot analysis of CysLTR2-L129Q in melanocytes. The 800-nm signal is the total receptor expression and the 700-nm signal is the α-tubulin loading control. Lane normalization factor (LNF) was calculated for each lane by dividing the 700 nm loading control signal intensity of the lane by the highest loading control signal intensity. Signals in the 800 nm channel were normalized (Norm signal) by dividing the signal intensity by the calculated LNF. The percent signal decrease was divided by the condition without drug and converted to a percentage.

			CysLTR2-L129Q						GFP		
		mock	no HP	10 nM HP	100 nM HP	1 µM HP	100 nM ent-HP	1 µM ent-HP	no HP	1 µM HP	1 µM ent-HP
Replicate 1	700-nm signal	604.00	559.00	660.00	831.00	532.00	504.00	423.00	465.00	566.00	482.00
	800-nm signal	-0.14	11.00	11.40	6.37	8.11	7.08	10.50	0.40	0.35	-0.20
	Norm signal	-0.20	16.30	14.30	6.37	12.70	11.70	20.60	0.72	0.51	-0.34
	Percent Signal Decrease		100.00	87.73	39.08	77.91	71.78	126.38	4.39	3.13	-2.08
Replicate 2	700-nm signal	6,230.00	7,830.00	6,040.00	6,280.00	7,450.00	8,230.00	8,920.00	8,420.00	10,900.00	8,260.00
	800-nm signal	-0.65	76.00	54.10	32.20	38.50	102.00	78.10	-0.12	0.04	0.34
	Norm signal	-1.15	106.00	97.80	56.00	56.50	136.00	95.70	-0.15	0.04	0.45
	Percent Signal Decrease		100.00	92.26	52.83	53.30	128.30	90.28	-0.20	0.06	0.59
Replicate 3	700-nm signal	13,600.00	3,920.00	2,430.00	2,300.00	560.00	601.00	999.00	852.00	3,380.00	713.00
	800-nm signal	-0.82	25.50	15.10	8.00	2.67	6.13	8.00	-0.27	-0.33	0.89
	Norm signal	-8.72	88.60	84.70	47.20	64.80	139.00	109.00	-4.28	-1.33	17.00
	Percent Signal Decrease		100.00	95.60	53.27	73.14	156.88	123.02	-4.83	-1.50	19.19
